# Using the COM-B model and Behaviour Change Wheel to develop a theory and evidence-based intervention for women with gestational diabetes (IINDIAGO)

**DOI:** 10.1186/s12889-023-15586-y

**Published:** 2023-05-15

**Authors:** Katherine Murphy, Jamie Berk, Lorrein Muhwava-Mbabala, Sharmilah Booley, Janetta Harbron, Lisa Ware, Shane Norris, Christina Zarowsky, Estelle V. Lambert, Naomi S. Levitt

**Affiliations:** 1 Department of Medicine, Chronic Disease Initiative for Africa (CDIA), University of Cape Town, Cape Town, South Africa; 2 Department of Human Biology, Division of Human Nutrition, University of Cape Town, Cape Town, South Africa; 3 Department of Paediatrics, MRC/WITS Developmental Pathways for Health Research Unit (DPHRU), University of the Witwatersrand, Johannesburg, South Africa; 4 South African Medical Research Council (SAMRC), Cape Town, South Africa; 5 Public Health Research Centre (CReSP – Centre de recherche en santé publique de l’Université de Montréal et du CIUSSS de Centre-Sud de Montréal), Montreal, Canada; 6 Department of Social and Preventive Medicine, School of Public Health, University of Montreal, Montreal, Canada; 7 School of Public Health, University of the Western Cape, Cape Town, South Africa; 8 Department of Human Biology, Division of Physiological Sciences, Faculty of Health Sciences, Research Centre for Health Through Physical Activity, Lifestyle and Sport (HPALS), University of Cape Town, Cape Town, South Africa

**Keywords:** Intervention development study, Behaviour Change Wheel, COM-B model, Women with gestational diabetes, Diet and physical activity intervention

## Abstract

**Background:**

In South Africa, the prevalence of gestational diabetes (GDM) is growing, concomitant with the dramatically increasing prevalence of overweight/obesity among women. There is an urgent need to develop tailored interventions to support women with GDM to mitigate pregnancy risks and to prevent progression to type 2 diabetes post-partum. The IINDIAGO study aims to develop and evaluate an intervention for disadvantaged GDM women attending three large, public-sector hospitals for antenatal care in Cape Town and Soweto, SA. This paper offers a detailed description of the development of a theory-based behaviour change intervention, prior to its preliminary testing for feasibility and efficacy in the health system.

**Methods:**

The Behaviour Change Wheel (BCW) and the COM-B model of behaviour change were used to guide the development of the IINDIAGO intervention. This framework provides a systematic, step-by-step process, starting with a behavioural analysis of the problem and making a diagnosis of what needs to change, and then linking this to intervention functions and behaviour change techniques to bring about the desired result. Findings from primary formative research with women with GDM and healthcare providers were a key source of information for this process.

**Results:**

Key objectives of our planned intervention were 1) to address women’s evident need for information and psychosocial support by positioning peer counsellors and a diabetes nurse in the GDM antenatal clinic, and 2) to offer accessible and convenient post-partum screening and counselling for sustained behaviour change among women with GDM by integrating follow-up into the routine immunisation programme at the Well Baby clinic. The peer counsellors and the diabetes nurse were trained in patient-centred, motivational counselling methods.

**Conclusions:**

This paper offers a rich description and analysis of designing a complex intervention tailored to the challenging contexts of urban South Africa. The BCW was a valuable tool to use in designing our intervention and tailoring its content and format to our target population and local setting. It provided a robust and transparent theoretical foundation on which to develop our intervention, assisted us in making the hypothesised pathways for behaviour change explicit and enabled us to describe the intervention in standardised, precisely defined terms. Using such tools can contribute to improving rigour in the design of behavioural change interventions.

**Trial registration:**

First registered on 20/04/2018, Pan African Clinical Trials Registry (PACTR): PACTR201805003336174.

**Supplementary Information:**

The online version contains supplementary material available at 10.1186/s12889-023-15586-y.

## Background

### Gestational diabetes mellitus

Gestational diabetes (GDM) is defined as diabetes, first diagnosed in the second or third trimester of pregnancy that is clearly not pre-existing type 1 or type 2 diabetes [1]. It is now one of the most common complications seen in pregnancy worldwide and is associated with several adverse pregnancy outcomes [2, 3]. While glucose levels usually return to normal after delivery, women affected by GDM face a significantly increased risk of developing type 2 diabetes (T2D), cardiovascular disease, hypertension and stroke in the longer term [4, 5]. In addition, through intrauterine exposure to maternal hyperglycaemia, their offspring are significantly more vulnerable to early onset obesity, T2D and cardio-metabolic disorders [6, 7]. A study in our setting found that within six years of a GDM pregnancy, 48% of the women followed up had progressed to T2D, more than two-thirds had three or more cardiovascular disease risk factors, and 27% of their children were overweight or obese [8].

In South Africa, the prevalence of GDM is estimated to be about 10–15% [9] but this is expected to grow, concomitant with the dramatically increasing prevalence of overweight/ obesity seen in women of reproductive age (currently at 60–70%) [10]. Given this expected increase and the high risk of progression to T2D among our local population, there is an urgent need to develop prevention interventions for our setting.

### Interventions for women with GDM

Lifestyle interventions focusing on diet, physical activity, glycaemic control and weight management are the primary therapeutic strategy for women with GDM [11, 12]. Reviews of the current evidence report that such interventions can enhance glycaemic control and weight management, reduce the risk of foetal macrosomia and post-partum progression to T2D [13–15].

Psychosocial wellbeing is a further important modifiable factor to target in such interventions. Women with a GDM diagnosis are more likely to develop clinically relevant levels of affective distress and antenatal and/or post-partum depression. Anxiety, stress and depression may also be an important causal factor in the development of GDM [16, 17]. In addition, there appears to be an important interaction between a woman’s psychological state and her motivation to follow a healthy lifestyle: post-partum depression is associated with an increase in comfort eating and decreased physical activity, whilst, conversely, physical activity reduces the symptoms of depression [18, 19]. Facilitating social support and building self-efficacy have been shown to be helpful strategies in improving behaviour change outcomes, including physical activity among women with recent GDM [20].

### Post-partum interventions for women with GDM

While post-partum screening for T2DM is recommended at 6 -12 weeks [21, 22], studies show that there is little emphasis on its importance in current models of GDM care and that attendance rates, even in high income countries are generally very low [23].Continuity of care from early in on the post-partum period, easy access to screening, reminders and risk awareness counselling are all factors that have been shown to improve screening rates and increase adherence [23–25].

It has been suggested that leveraging scheduled ‘well-baby’ visits at health services, including the child’s vaccination program and follow-up, may provide a good opportunity to conduct the necessary tests and provide follow-up advice to GDM mothers [26, 27].

While women are usually highly motivated to change their behaviour during pregnancy to safeguard the baby, motivation to sustain a healthy lifestyle tends to decrease significantly once the baby is born. Women cite the demands of childcare, work commitments, tiredness, diminished social support, difficulty in balancing household demands and expectations, finances and resources as barriers to maintain the changes in diet and physical activity that they initiated while pregnant [24, 27, 28]. In addition, factors in the broader built, food, and social environments in which women live can make it difficult for them to sustain lifestyle recommendations [27, 29, 30]. Despite these challenges, women are receptive to longer-term support and post-partum interventions have been shown to be effective in reducing the incidence of diabetes or delaying its onset among women who have had a GDM pregnancy [31]. It is recommended that interventions provide post-partum screening along with information on the long-term risk of progression to type 2 diabetes, social and professional support for sustaining a healthy lifestyle, counselling on the advantages of breastfeeding and enlist the direct involvement of partners and family members [13, 14, 25, 27].

The evidence on interventions for GDM women is still limited and more high-quality intervention trials, which extend from pregnancy into the post-partum period, study long-term outcomes for both mother and child and measure cost-effectiveness, are urgently needed to build a robust evidence base [14, 32]. A further limitation of the current evidence is that most of the existing research has been conducted in high-income countries and the value of lifestyle interventions has not yet been established among women living in low-to-middle income countries [13, 14, 25]. Research is needed to understand how to effectively engage and support women in these settings to modify their lifestyle behaviours and mitigate the risks of GDM and T2D.

### The IINDIAGO intervention study

The IINDIAGO study is an exploratory, intervention trial (PACTR2018050033 36,174, Pan African Clinical Trials Registry (PACTR)) which aims to develop and evaluate an integrated health system intervention to reduce the risk of the adverse effects of GDM during pregnancy and of progression to T2D post-partum among disadvantaged GDM women attending three large, public-sector hospitals for antenatal care in Cape Town and Soweto, South Africa.

The exploratory trial was completed in 2021. Study participants are 370 women diagnosed with GDM between 24–36 weeks gestation from urban communities of lower socio-economic status with a disproportionately high burden of T2D. The intervention includes antenatal and postnatal education and counselling from trained peer counsellors and a diabetes nurse, as well as referral and follow up for diabetes screening at 6 weeks post-partum. This is offered at the convenient location of the Well Baby clinic, where mothers routinely bring their children for their first immunisation. The main outcomes of this trial are the completion of the 6-week post-partum oral glucose tolerance test (OGTT) and reduction in diabetes risk at 12 months post-partum, using a composite measure of weight, waist circumference and dysglycaemia.

The development of the IINDIAGO trial follows the UK Medical Research Council’s (MRC’s) recommended 4 stages of research for the development and evaluation of complex health interventions (develop-pilot-evaluate-implement) [33]. Whilst the MRC framework emphasises the importance of basing intervention design on a theoretical understanding of the target behaviour/s and how the intervention could potentially cause behaviour change, it does not offer detailed guidance on the intervention development stage (stage 1). We identified the Behaviour Change Wheel (BCW) [34] as helpful in meeting our need for more comprehensive and detailed guidance on how to design behaviour change interventions.

This paper reports in detail on the process involved in the development of a theory-based behaviour change intervention for GDM women, prior to its preliminary testing for feasibility and efficacy in the health system (stage 2 of the MRC Framework).

## Methods/design

### Theoretical framework for intervention development

In developing the intervention, we followed the step-by-step guidance of the the Behaviour Change Wheel (BCW) [34]. This framework synthesises key theoretical constructs from 19 different frameworks in the behavioural science literature and links them to a theoretical model of behaviour change -the COM-B- that is sufficiently broad to be applied to a diversity of behaviours across different settings. It also provides a standardised taxonomy for characterising interventions and drawing the possible relationships between outcomes and mechanisms of change, which is regarded as a significant advance in the discipline as it facilitates the process evaluation, replication and comparison of interventions [34, 35].

### The COM-B model

**Fig. 1:**
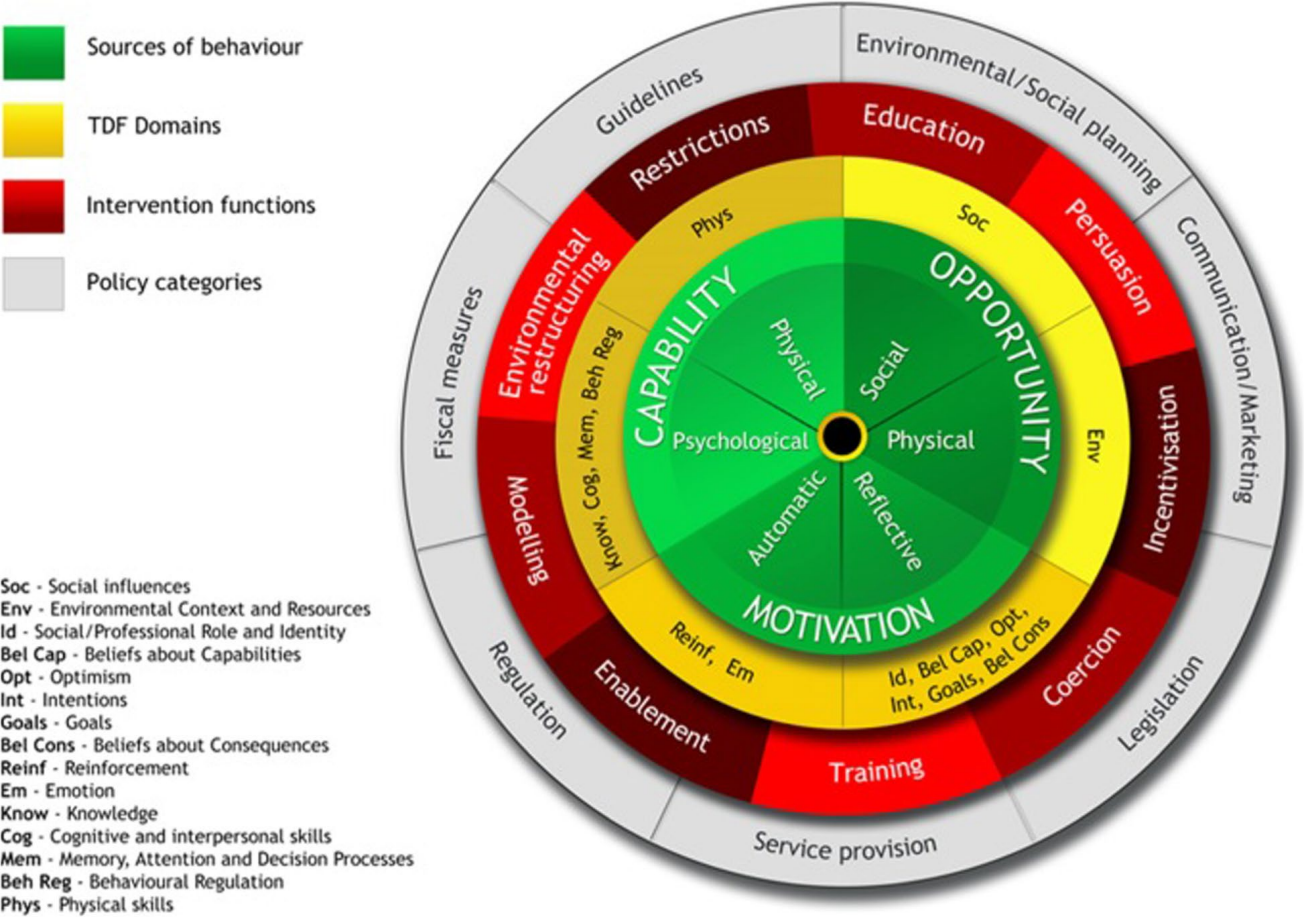
The Behaviour Change Wheel (BCW)

As illustrated in Fig. 1 the BCW consists of 4 layers. The hub of the wheel consists of the COM-B model, which posits that changing any behaviour of an individual, group or population involves changing one or more of the following: *Capability, Opportunity* and *Motivation*. *Capability* can be physical or psychological; *Opportunity* refers to factors in the physical or social environment which facilitate, or hinder behaviour change and *Motivation* can be reflective (involving conscious evaluations and planning) and/or automatic (unconscious emotional responses, impulses or habits).

### Theoretical Domains Framework (TDF)

Within the three COM-B components that generate the behaviour, it is possible to further distinguish *Capability*, *Opportunity* and *Motivation* using the Theoretical Domains Framework (TDF) (the layer surrounding the COM-B hub in Fig. 1). *Capability* is further broken down into knowledge; skills, memory, attention and decision-making processes and behavioural regulation; *Opportunity* into environmental context and resources, and social influences; and *Motivation* into social/professional role and identity, beliefs about capabilities and consequences, optimism, intentions, goals, reinforcement and emotion.

### Intervention functions and policy options

Surrounding the COM-B model and TDF is a layer of 9 intervention functions to choose from. Finally, the outer layer of the wheel identifies 7 types of policy options that one can use to deliver the intervention functions. As the behavioural related components of the COM-B model can be addressed through more than one intervention function and policy category, the inner wheels can be ‘moved around’ the centre core of the BCW.

## Application of BCW method to IINDIAGO

**Fig. 2:**
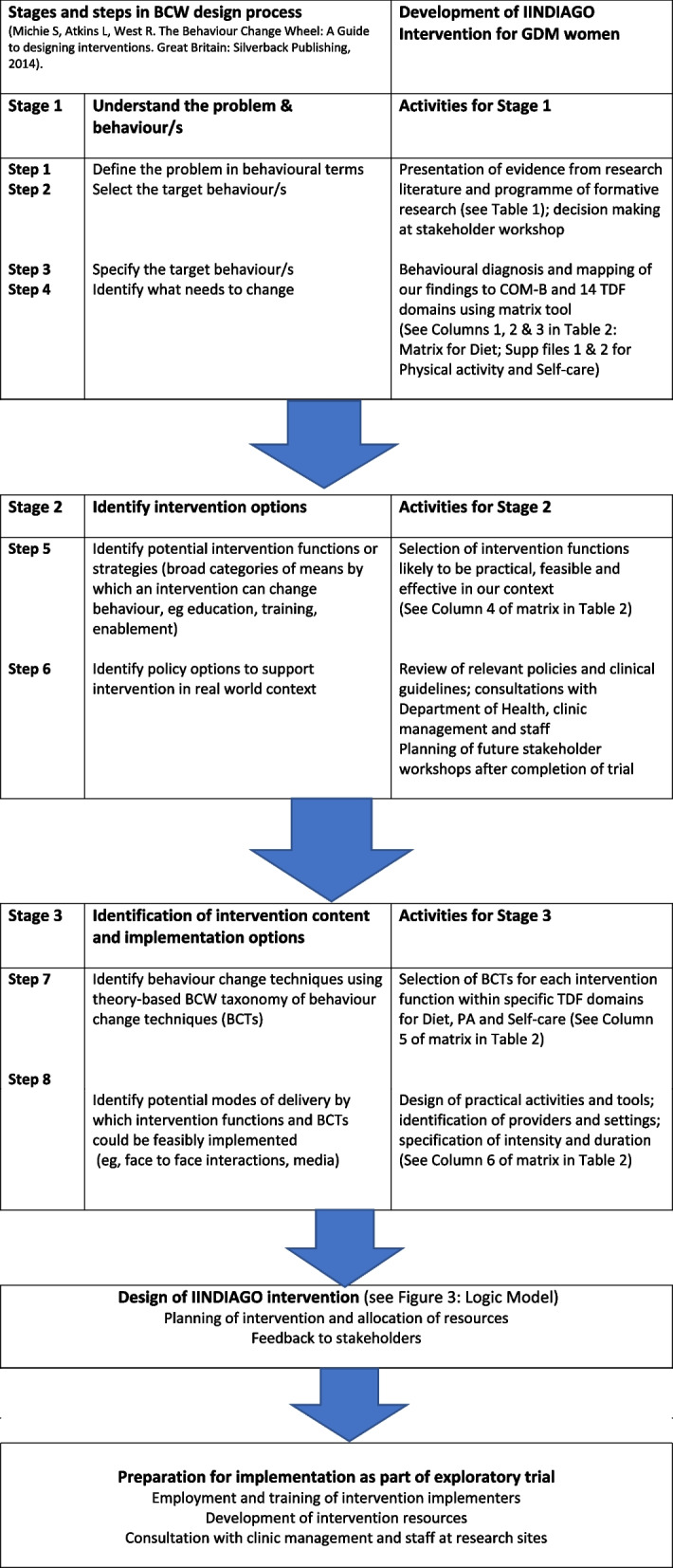
Flow diagram for intervention design process

We followed the BCW’s systematic step-by-step process for intervention development, starting with a behavioural analysis of the problem and making a diagnosis of what needs to change, and then linking this to intervention functions and behaviour change techniques to bring about the desired change (see Fig. 2: Flow diagram for design process).

### Formative research and stakeholder consultation

Prior to beginning the development process, we undertook a review of the relevant research literature to identify the behavioural, health system and broader environmental determinants for GDM and progression to T2D, and what types of health system interventions have proven viable and effective. In addition, to understand and define the problem of GDM in our setting and the barriers and facilitators to behaviour change relevant to our target population, we undertook a programme of formative, context-driven information gathering (see Table 1). This programme of research took about 2 years to complete.

**Table 1:** Programme of primary data collection to understand context, target group and potential implementers

A document review of all existing policies and clinical guidelines for the management of GDM women in local public sector health services [36]
Individual, in-depth interviews with key informants and stakeholders, including Department of Health managers, directors, clinicians and policymakers to investigate current practice and the potential for and acceptability of an intervention [36]
Nine focus groups and five in-depth individual qualitative interviews with GDM women (*N* = 35) sampled from our target population of women attending the GDM clinics at Mowbray Maternity and Groote Schuur hospitals in Cape Town. The aim of this study was to gain insight into women’s experiences and views of GDM, the feasibility of lifestyle modification in their context, their perceived needs in relation to GDM care and their opinions on a potential intervention [37]
A cross-sectional study on the dietary intake and beliefs of 239 pregnant women with GDM receiving care in a Cape Town public sector hospital. Dietary intake was assessed using a quantified Food Frequency Questionnaire and beliefs relating to food choices were assessed using the Theory of Planned Behaviour (TPB)[38]
Thirty qualitative interviews with key informants, healthcare providers and pregnant women to assess the acceptability, feasibility, and potential for the integration of GDM follow up care into primary care at a local and district level [36, 39] and to identify the lessons learnt from South Africa’s successful integration of Prevention of Mother-to-Child (PMTCT) programmes into primary care services [40] **Ethical approval** Written informed consent was obtained from each participant in the focus groups and interviews. Ethics approval for the research was obtained from the University of Cape Town (HREC: 946/2014) and Université de Montréal (CR CHUM: 2018–7091, 17.128-ID). Permission to conduct the folder audit was obtained from the relevant hospital authorities

Findings from these studies were presented for discussion and further interpretation at a stakeholder workshop, which included the IINDIAGO team, clinicians, dieticians, physical activity specialists and representatives from the department of health. Recommendations from this workshop, findings from the formative studies and the literature, and the combined experience and multidisciplinary expertise of the IINDIAGO project team all served to guide and inform each of the ensuing steps- in the development process.

## Step-by-step development of the intervention

### Stage 1: Understand the problem and behaviours

Steps 1 and 2 were undertaken at the stakeholder workshop mentioned above. This involved identifying the specific behaviours that need to be changed to address the problem of GDM, specifying the people and settings involved, and then selecting those behaviours that were most likely to impact on the problem, and that were feasible to target in a health system intervention.

In Steps 3 and 4 two members of the research team (KEM and JB) used the COM-B model and the Theoretical Domains Framework (TDF) to map out a detailed analysis of the selected target behaviours in their context and to identify what precisely needed to change in GDM women to achieve the desired change in behaviour. This was facilitated by having analysed the findings of our research with GDM women on the factors which hinder or facilitate lifestyle change during and beyond the GDM pregnancy, according to the main categories of the COM-B model: Capability; Opportunity and Motivation [37].

### Stage 2: Identify intervention options likely to achieve desired behaviour change

In Step 5, we considered intervention functions for each of TDF domains with reference to the nine options in the BCW [34]. We then selected the intervention functions or strategies likely to be practical and feasible in our context and effective in bringing about the desired behaviour changes in GDM women.

Step 6 involves considering potential policy options that could support the implementation of the intervention in the real-world context. The documentary review (See Table 1) was conducted to understand the current policy framework. Future policy and implementation options are to be fully considered, along with stakeholders, once all the findings of the exploratory trial are available.

### Stage 3: Identify content and implementation options

We then moved on to establish the content of the intervention (Step 7) and identify possible modes of delivery (Step 8). For this task, we drew on Michie’s comprehensive, evidence-based taxonomy of behavioural change techniques (BCTs). This provides a standardised language with precise definitions for describing the active ingredients of interventions which are designed to bring about changes in the target behaviours [34]. We also used the table which links the most frequently used and effective BCTs to specific intervention functions. In Step 8 we considered which modes of delivery were relevant, practical, and feasible, given the limitations of our setting and resources.

Whilst this detailed mapping process, incorporating theory, evidence and practical considerations was conducted by KEM and JB, it was presented periodically for review by the larger project team (all other authors) before the intervention design was finalised.

## Results

### Stage 1: Understand the problem and the behaviours

#### The problem

A description of the various dimensions of the problem has been reported in several other papers from the IINDIAGO project [36–38, 41, 42]. One of these, presents the full behavioural diagnosis of the focus group findings using the COM-B model [37]. For the purposes of this paper, the key findings and their implications for designing the intervention are summarised below:

In our setting, women diagnosed with GDM at the level of primary care are referred to a secondary or tertiary level hospital for the remainder of their antenatal care [36]. Here, they typically receive a high level of clinical care, with positive birth outcomes. In contrast, they receive little attention postpartum, despite the national guidelines recommending a 6-week post-partum follow-up. Several barriers impede follow up: (i) little emphasis during antenatal care on postpartum GDM risks; (ii) an absence of a standardised and structured post-partum care pathway to ensure the provision and accessibility of post-partum screening for diabetes; and (iii) the inconvenience of women having to navigate separate clinics for herself and her baby post-partum. Both key informants [36] and women [37, 41] agreed that the lack of post-partum follow-up was an important gap in the existing model of care. They supported a proposal to combine the scheduled 6-week immunisation visit for the infant with a post-partum OGTT for the mother at Well Baby clinics for improved accessibility and convenience. This strategy aligned well with current Department of Health policy initiatives, which emphasise integrated care for the mother: baby dyad.

Our focus group interviews with women showed that while they were satisfied with the standard of clinical care, they were frustrated with the limited information they had received at the time of referral and enrolment at the GDM clinic [37]. Many women reported experiencing intense anxiety about the GDM diagnosis and were frustrated by the lack of opportunity to ask questions or raise their concerns with antenatal care providers at this time [37, 41]. They believed that the emotional and psychological burden of a GDM pregnancy was not adequately recognised by antenatal care providers, whose primary focus was the safety of the baby [41]. Women who participated in the focus groups felt that their emotional and psychological needs could be partly met through similar facilitated discussions, where they could share their experiences and feel less alone [42].

Typically, women received a single, brief consultation immediately after diagnosis with a dietician, who advised them to follow a restrictive diet and lose weight. The only information resource they received was a one-page diet sheet with a brief explanation of GDM [36, 37]. They unanimously felt this to be insufficient and expressed a strong desire for more in-depth information on the risks of GDM and for supportive counselling on healthy eating, which acknowledged the difficulties they faced in changing their dietary behaviour and in losing weight. These included the expense of healthy foods; a lack of knowledge and skills for how to prepare healthy meals; the difficulty of eating differently from the family and pregnancy related cravings for foods high in fat or sugar. Women’s knowledge of the role of physical activity in improving glucose control was very limited and its importance appeared to be rarely mentioned by healthcare providers. Whilst women were generally motivated to make dietary changes to protect the unborn baby, their commitment to change did not extend beyond the pregnancy. A critical finding was that many women were entirely unaware of their increased risk for the development of T2D post-partum or the possible risk of metabolic disease for their child. They also reported that sustaining dietary changes made in pregnancy was very difficult because intrinsic motivation and social support from the family diminished once the baby was born.

These research findings offered us clear direction as to how to tailor an intervention to meet the needs of our intended target group. Key objectives included that our intervention: commence around the time of the GDM diagnosis when women reported experiencing heightened anxiety and confusion; incorporate a strong element of psychosocial support to alleviate distress and help women cope with their fears around the GDM pregnancy; address the health system’s failure to provide convenient and accessible services for the 6-week post-partum OGTT and to emphasise the importance of this screening in detecting risk for progression to T2D; attend to women’s evident need for more information on GDM, as well as their need for knowledge, skills and resources for sustained behaviour change during and after pregnancy; and consider involving the family, as family expectations for the woman to revert to a ‘normal’ diet were reported as a significant obstacle to sustaining lifestyle change post-partum. These findings are virtually identical to those reported by the Living study, which conducted formative qualitative research with women in India and Bangladesh for the purposes of developing a post-partum intervention [27], suggesting that many of the recommendations for interventions made in current reviews are also relevant to low-and-middle income countries [13, 14, 24, 25, 32, 43]. Findings that have to date, received less emphasis in the current literature included that our intervention needed to clearly re-frame current messaging around GDM so that it is understood to be a warning sign of increased, long term risk for T2D for both mother and baby, and not just a pregnancy related health issue; to offer psychological support to women who struggle with feelings of guilt and shame when they fail to adhere to the intensive regime of care and to follow the strict dietary prescriptions; and to incorporate opportunities for peer interaction, support and problem solving, which was expressed as a strong desire in our focus groups. Furthermore, our findings highlighted the importance of us developing information materials and messaging about lifestyle change that is acutely sensitive to the poor socio-economic circumstances of our target population of GDM women and their families, which severely constrains their choices. Women’s negative attitudes to the traditional, prescriptive advice-giving approach to health education in our health services indicated strongly that we use methods of education and counselling that are patient-centred, i.e. that support autonomy in decision making, encourage women to find their own solutions, and build self-efficacy and optimism.

#### The behaviours

The selected target behaviours for the intervention were the main, modifiable risk behaviours associated with the development of GDM and progression to T2D, namely diet and physical activity. Self-care was added as a novel third target behaviour to incorporate the aspect of psychological well-being and convey a more holistic, woman-focused health message.

The exhaustive analysis of these target behaviours (Steps 3 & 4 of the BCW) according to COM-B model and TDF, is illustrated in Table 2 in relation to ‘Diet’. The equivalent matrices for Physical Activity and Self-care are included as additional files (see Supplementary Files 1 and 2).

**Table 2:** IINDIAGO MATRIX: Expanded COM-B: TDF domains, theoretical constructs and relevance to GDM women (identified barriers & enablers): DIET

**CAPABILITY Psychological**	**Formative Assessment**	**For behaviour change to occur, women with GDM would need to:**	**Intervention Function: What needs to be done to change behaviour**	**Behaviour change techniques (BCTS)**	**Resources, tools and activities**
**KNOWLEDGE** **(Awareness)** ***Do you know about X?***	• Not well informed about the relationship between GDM andT2D; what constitutes a healthy diet for (self, kids, T2D diabetics); role of diet in T2D • Have general misconceptions about healthy eating and body size • Health care providers (HCP) give inconsistent or confusing messages/info	**Increase knowledge of** • GDM/T2D; foetal programming; role of diet in developing T2D, potential for prevention through lifestyle changes • what constitutes a healthy diet for self, kids, GDM • role of portion control especially during pregnancy (eating for 2 myth) • role of glucose and insulin • how to sustain a healthy diet through life course	**EDUCATION** • About ways of enacting desired behaviour and avoiding undesirable one • Need to provide credible, appealing info to take home • Clear, consistent, standardised messages • Info needs to address prevalent misconceptions about healthy eating and dispel myths	5.1 Information about health consequences	• Brochure on Diet Leaflet on diabetes • Individual counselling, peer group discussion • Visual aids for raising awareness of sugar and fat content (coke, fruit juice etc.- produce kit for the counsellors) • Visual aid for appropriate weight gain for baby/child
**Cognitive and Interpersonal Skills** **(Ability acquired with practice)** ***Do you know how to do X?***	• Lack skills in purchasing of healthy foods • Don’t know how to control portions • Lack skills to negotiate or challenge social and family norms	**Acquire skills** • to purchase healthy food; to read food labels and to exercise portion control • to change family diet and/or persuade family to change their diet • to plan healthy meals	**TRAINING** • Provide tools to understand food labels to use when shopping • Demonstrate portion control • Provide skill training and practice to improve communication skills • Provide tips for planning healthy meals	4.1 Instruction how to perform behaviour 15.2 Mental rehearsal of successful performance	Diet brochure (food labels graphic and portioned plate graphic) • Recipe book • Peer group workshop ‘agent for change’ • Activity: problem solving strategies for negotiating change • Home visit to engage family • Meal planning tips pg 6 of Recipe book
**Memory, Attention, decision, processes** **(Retain, focus on info, make decisions)** **Is X** ***something you usually do?*** ***Do you actually do X in your context/situation?***	• Lose focus on healthy diet once pregnancy is over • Need reminders to apply knowledge about diet in real life settings to make better choices	• focus on diet and make healthier decisions and choices • pay more attention to diet on a long-term basis • translate knowledge about diet to actually making healthy food choices/decisions	**TRAINING** • Identify opportunities to practice making healthier decisions in different settings (eating out; supermarket; work; home) • Assist woman to make realistic appropriate decisions for long term in their context • Provide social support (peers, PC) and feedback	6.1 Demonstration of the behaviour 2.2 Feedback on behaviour 3.2 Social support 8.2 Behaviour substitution	Diet brochure: (pg10 Label tool for the purse as reminder, pg3 plate model for fridge) • Place mat colouring activity for kids • Peer group workshop ‘making healthy choices’ • Supermarket corner activity • Analysing sample menus
**Behavioural regulation** **(Anything aimed at managing or changing objectively observed action)** ***Do you have systems or tools for monitoring whether or not you have carried out X?***	• Women do not have tools to monitor dietary change and weight • Dietary monitoring ends after pregnancy (self and HCP) • Misconceptions about food cravings in pregnancy result in lapses in dietary regulation	• improve capacity to monitor dietary behaviour • set goals and review progress about diet during and after pregnancy • use healthy foods to manage pregnancy cravings • recognise signs and symptoms of hypo/hyperglycaemia and use healthy foods to self-manage	**ENABLEMENT** • Provide opportunity, support and tools to self-monitor dietary behaviour or habits • Offer strategies for glucose regulation and managing pregnancy cravings using healthy foods • Provide continuation of care—ongoing follow up through counselling	2.3 Self-monitoring of behaviour 2.4 Self-monitoring of outcomes (Method to record outcome) 2.6 Biofeedback (not just weight) 1.5 Review of behaviour goals (jointly with HCP)	• Reviewing food shopping receipts, competition as part of ‘making healthier choices workshop’ • Supportive counselling: Negotiated joint review of progress about dietary change Diary (to record details about food, energy, sleep, physical symptoms, attitude, motivation) • Scale available at peer meetings
**CAPABILITY** **Physical**	**Formative Assessment**	**For behaviour change to occur, women with GDM would need to:**	**Intervention Function: What needs to be done to change behaviour**	**BCTS**	**Resources/Tool and activities**
**Physical Skills** ***Do you have the physical skills necessary to do X?***	• Lack adequate cooking skills for healthy foods	• acquire the physical skills to prepare foods using healthier ingredients and methods	**TRAINING** **• p**rovide guidelines and recipes **MODELLING** provide cooking demonstrations	4.1 Instruction on how to perform behaviour 6.1 Demo of behaviour	• Cooking demonstrations, chopping methods • Recipe books with instructions on how to improve family meals using healthier methods
**MOTIVATION** **Reflective** ^**a**^	**Formative Assessment**	**For behaviour change to occur, women with GDM would need to:**	**Intervention Function: What needs to be done to change behaviour**	**BCTS**	**Resources, tools and activities**
**Social role and identity** ***Is doing X compatible or in conflict with your identity?***	• Women prioritise their role /identity as mothers and as carers for family • Do not fully recognise the compatibility of their identity as mothers and their potential influence on the diet of family	• Personally identify with a healthy lifestyle to be a confident example to their family • Feel that managing dietary behaviours of the family is an important part of their maternal role	**PERSUASION** • Highlight compatibility with current identity, but expand it to include NCD prevention Emphasise role of mother as change agent and importance of role modelling for kids	5.2 Salience of health consequences 13.2 Reframing 13.1 Identification of self as role model 13.5 Identity associated with changed behaviour	• Facilitated peer group discussion (being a change agent in family) • Value exercise – explore value related to self-identity • Affirm personal strengths and identity as part of change strategy in counselling
**Beliefs about capabilities** **(acceptance of the truth, reality or validity about ability** ***How difficult or easy is it to do X?***	• Women feel insufficient sense of agency about food choices • Not confident to maintain dietary change after delivery in the long term	• Believe they are capable of change despite constraints • Feel able to make healthier choices for themselves and family. (greater agency and locus of control) • Believe that healthier eating can be a sustainable lifestyle	**PERSUASION** • To enhance self-efficacy and self-monitoring **ENABLEMENT** • Assist problem solving to address overcoming context specific barriers • Facilitate activities that promote self-efficacy	3.1 Social support 6.1 Modelling 1.2 Problem solving 15.3 Focus on past success	• Non-directive individualized counselling to enhance self-esteem, confidence and self-autonomy • Confidence scale tool in one-to-one counselling to promote change talk • Peer group theme “overcoming barriers” to address problems/barriers in group setting
**Optimism** **(confidence that goals will be achieved)** ***How confident are you that you can overcome/manage X?***	• Believe T2D/ GDM is mainly related to family history i.e., their fate • Lack sense of agency/ fatalistic • Felt very anxious and scared at diagnosis	• Need to feel optimistic that they can prevent progression to T2D with dietary adjustment • Establish a new perspective and aspire to emulate others who have achieved dietary change • Feel optimistic that dietary change for themselves and family is possible	**ENABLEMENT** • Build optimism in capability to change diet **EDUCATION** • Need to dispel belief that T2D is inevitable and related to family history and it is irreversible **PERSUASION** • Convince women that dietary change can prevent/reverse negative health outcomes	3.3 Social support emotional 15.1 Verbal persuasion about capability 13.2 Reframing 6.2 Social comparison	• Non-directive counselling approach will build optimism and self-efficacy • Affirmation and acknowledgement of small changes in counselling • Testimonials in IC4H • Peer modelling success stories as part of peer group discussion “Overcoming barriers”
**Beliefs about** **Consequences (acceptance of reality validity of outcomes about behaviours)** ***What do you think will happen if you do X?***	• Underestimate personal dietary risk and association between GDM/T2D and diet • Believe consequences related to pregnancy only • Do not extend beliefs about consequence to the family • Negative beliefs about healthy foods (that food is tasteless or boring; or the health eating will leave them socially isolated, hungry, and will affect family relationships) • Negative beliefs based on experience of hospital food	• Assess risk more accurately • Make clear link between behaviour and health outcomes • Believe GDM = both a warning sign that diet is unhealthy and a red flag for future risk • Believe in benefits of healthy diet for self and family • Believe healthy eating can be sustainable, palatable and affordable	**EDUCATION** • Provide tools to assess risk that can be shared with family to change **PERSUASION** • Reframe what GDM signifies for self and family **MODELLING** • Provide strategies to deal with potentially negative consequences of eating healthily	5.1 Information about health consequences 13.2 Reframing 6.1 Demonstration of the behaviour 8.1 Behaviour practice/rehearsal	• Food quiz in IC4H brochure to assess diet of self and family • Dispelling of myths related to risk and address beliefs/attitudes about healthy eating (Peer group “Overcoming Barriers” • Cooking demo with emphasis on attractive presentation
**Intentions** **(A conscious decision or resolve to act)** ***Have you made a decision to do X (long term)?***	• Intentions during pregnancy differ to those pre-pregnancy or post- partum • Commitment is short term i.e., during pregnancy only • Intentions to change dietary behaviour seen as only necessary for self/mother not the larger family	• form long term intentions after pregnancy (Stability of intentions) to change diet for self and family • expand intentions to include diet of partner and children	**ENABLEMENT** • Encourage and support in formulating intentions to change diet for self and family in long term	9.2 Pros and Cons 9.3 Comparative imagining of future outcomes	• Assessing readiness to change. (use 1–10 Motivational Interviewing scale in individual counselling) • Decisional balance sheet used as an interactive peer activity in workshop “overcoming barriers” and individual counselling
**Goals** **(Outcomes that individual wants to achieve)** ***How much do you want to do X?*** ***[What exactly are you going to do?]***	• Don’t set goals beyond pregnancy • Lack support and guidance in realistic goal setting	• Set goals for dietary change for self and family (SMART and personalised) • Set small achievable, and interim goals	**ENABLEMENT** • Provide support and guidance for realistic goal setting • Affirm small interim goal setting • Prompt planning during and after pregnancy	1.3 Goal setting (outcome) 1.1 Goal setting (Behaviour) 1.9 Commitment 8.7 Graded tasks 1.4 Action planning	• Non-directive counselling approach to define personalized goals • R2H Card/ health care case record • Group game for promoting commitment: Group chooses a recipe a week to try out or adapt own recipe and give feedback in peer groups
**MOTIVATION** **Automatic** ^**b**^	**Formative Assessment**	**For behaviour change to occur, women with GDM would need to:**	**Intervention Function: What needs to be done to change behaviour**	**BCTS**	**Resources/Tool and activities**
**Reinforcement** **(Rewards, incentives, sanctions, punishments, consequences, contingencies)** ***Are there any incentives to do X?***	• Currently have healthier eating habits during pregnancy followed by a relapse post-partum (baby = incentive) • Entrenched unhealthy eating habits or patterns	• Substitute unhealthy eating habits with healthy ones • Develop strategies which will help in establishing new habits and patterns	**TRAINING** give tips on environmental restructuring to aid habit formation and habit reversal	8.3 Habit formation 8.4 Habit reversal	• Explore incentives/rewards for change pp (agent of change workshop) • Offer prompts and tips on how to develop habits (e.g. association, repetition) for replacing unhealthy behaviour with alternate behaviour • Prompt thinking about how to restructure home environment to enforce new healthy habits (e.g. Cold water in the fridge, no salt on table, available healthy snacks and visual cues) • Repeated stimulus in affirming health foods to kids
**Emotion** **(A complex reaction pattern for dealing with event or issue)** ***Does doing X evoke and emotional response?***	• Experience negative emotions when diagnosed (anxiety, feeling overwhelmed) • Experience negative emotions related to the challenge of having to change their diet (feelings of isolation, failure, guilt, shame) • Feelings of stress can result in unhealthy eating patterns • Identity, culture and tradition play a role in dietary choices and perceptions of body shape	• Associate positive emotions with healthy eating and healthy foods • Understand relationship between stress and unhealthy eating patterns • Develop more positive emotional responses to challenges of dietary change	**PERSUASION** • Help woman deconstruct emotional associations with food **ENABLEMENT** • Provide a supportive environment for women to explore emotional responses to the prospect of dietary change create a safe space for a conversation about dietary change without invoking the success/failure paradigm or stigma	5.6 Information about emotional consequences 3.3 Social support (emotional) 5.4 Monitoring of emotional consequences 13.2 Persuasion reframing	• Activity around deconstructing emotional eating and cravings in peer group “making healthier choices” • sensitization of counsellors regarding emotionally loaded words or terms around diet and weight
**OPPORTUNITY** ***Environmental/social***	**Formative Assessment**	**For behaviour change to occur, women with GDM would need to:**	**Intervention Function: What needs to be done to change behaviour**	**BCTS**	**Resources, tools and activities**
**Physical Environmental** **Context and resources** ***To what extent do physical or resource factors facilitate or hinder X?***	• Time is perceived as a barrier • Perception that healthy foods are too expensive, yet spending on take away and processed food (e.g., KFC) • Have the means to prepare healthy food at home (i.e., kitchen equipment) • Experience high exposure to promotion of unhealthy choices and relatively little exposure to healthy eating messages	• Learn and practice how to prepare healthy recipes quickly and efficiently • Learn how to eat healthily on a budget by reallocating money spent on take-away • Have critical understanding of the obesogenic environment • Have access to information and resources that promote healthy eating	**TRAINING** • Strategies to eat healthily on a budget **ENABLEMENT** • Increase critical awareness of ads marketing unhealthy foods • Provide resources and information that can serve as cues/reminders for healthy choices	6.1 Demonstration of the behaviour 4.1 Instruction on how to perform the behaviour 13.2 Reframing 12.5 Adding objects to environment	• Cooking prep and demos which do not cost more than usual • Provide recipe book as a take home resource • Facilitated discussion with peer group in workshops “*Overcoming barriers and Making healthy choices*” • Activity – deconstruct ads • Recipe books and brochure • Posters/visuals in peer group venue to reinforce healthy messages
**Social Influence** **(social pressure, norms, support, power relations, group identity, modelling)** ***To what extent do social influences hinder or facilitate X?***	• Women receive good support during pregnancy, less pp from partners, family. HCPS and other GDM women • Believe GDM is the mother’s issue only • Post-partum—no longer have support from HCPS and other GDM women and family • Experience social pressure to conform group identity eating habits • Enjoyed peer interaction of focus group, wished they had such support during pregnancy	• Continued social support in pp period from HCPS, GDM women, partners and family • Perceive social norms supportive of change believe others are doing it to feel less isolated • Relatedness- feel more support from HCPS, believe that they care about preventing progression to T2D and value their health • Understand GDM as a family issue	**ENABLEMENT** • Require health care system follow up post-partum • Family and friend invited to peer group and counselling sessions • Reframe GDM as whole family issue **MODELLING** • Provide testimonials in materials (success stories) to promote relatedness	3.3 Social support 3.1 Unspecified 13.2 Reframing 6.2 Social comparison	• Peer group and home visits establish continuity of care • Lay counsellors to use non-directive counselling style to address power imbalance and promote relatedness • Group sharing of recipes, cooking methods can promote feelings of social support • Choose recipes for a week. Everyone gives feedback on experience

^a^Reflective motivation (higher order cognitive processes, part of self-determination and self-regulation)

^b^Automatic motivation (Reactions driven by unconscious internal processes e.g. habits, drives, desires, impulses, inhibitions).

As can be seen in Table 2, we mapped out an analysis for each of the 14 TDF theoretically derived domains within the broader categories of the COM-B model: Capability; Opportunity and Motivation. Column 1 also includes exemplar interview questions from the BCW guide, which we used as a tool to interrogate our knowledge of our target audience in relation to each TDF domain. Column 2 documents the findings from our formative research with GDM women for every TDF domain, illustrating how our behavioural diagnosis was grounded in our formative research findings. This column shows the numerous barriers that we found GDM women in our setting experienced in managing GDM and changing their behaviour to prevent progression to T2D. Column 3 identifies what needs to change in GDM women (and in their social and physical environment) to enable them to achieve the desired change in behaviour change. These become the potential targets for intervention.

### Stage 2: Identify intervention functions and supportive policies

The BCW provides a range of nine candidate intervention functions that have been found to be effective in bringing about behaviour change: education; persuasion; incentivisation; coercion; training; restriction; environmental re-structuring; modelling and enablement. Our selection of potential intervention functions or strategies for each COM-B component and TDF domain for Diet is illustrated in Column 4 of Table 2. Several intervention strategies were often relevant to one domain.

Step 6 For the purposes of implementing the intervention in the health system, we consulted with the Department of Health and the clinic staff and management at the research sites to secure their support and cooperation. The documentary review and our earlier interviews with policymakers and healthcare managers and providers, (see Table 1), showed that our planned intervention would be acceptable and feasible within the framework of existing policies and clinical guidelines for GDM care and that the routine services could accommodate the types of intervention activities we were planning.

### Stage 3: Identify content and implementation options

Column 5 in Table 2 illustrates the selected BCTs for each intervention function within specific TDF domains for “Diet” (Physical Activity and Self Care, see Supplementary files 1 and 2).

Column 6 in Table 2 illustrates the practical activities, the tools and resources by which the BCTs and intervention functions could be feasibly delivered, and the intensity and duration of intervention. For example, we planned for the intervention functions of *Enablement; Education* and *Persuasion* and their associated BCTs*: social support; verbal persuasion about capability; reframing and social comparison,* to be delivered through the activities of education and counselling by lay counsellors, peer support groups and the use of real-life testimonials in the health education leaflets, which modelled people in similar circumstances overcoming commonly perceived obstacles (see Supplementary File 3 for more information on the resources for the intervention).

In summary, Table 2 illustrates how we progressed through the various steps in the BCW to tailor our intervention to our target population: starting with a detailed, behavioural analysis of the evidence we had gathered on GDM women in our setting (Column 2), informed by the COM-B and TDF (Column 1); then proceeding to make a behavioural diagnosis of what GDM women needed to do to change their behaviours (Column 3); to identifying what intervention functions or strategies could enable them to overcome the identified barriers and achieve the desired behaviour change (Column 4 & 5); and finally, to considering what intervention activities and resources could potentially deliver such objectives, whilst being appropriate, acceptable and affordable (Column 6). The matrices were a helpful tool which we devised to combine all the different elements we needed to consider for our intervention to be effective in enabling change in each of the three selected target behaviours. These comprehensive ‘maps’ were then used to make decisions about the active ingredients and the scope of intervention within the limits of our available resources.

## Overview of planned IINDIAGO intervention

**Fig. 3:**
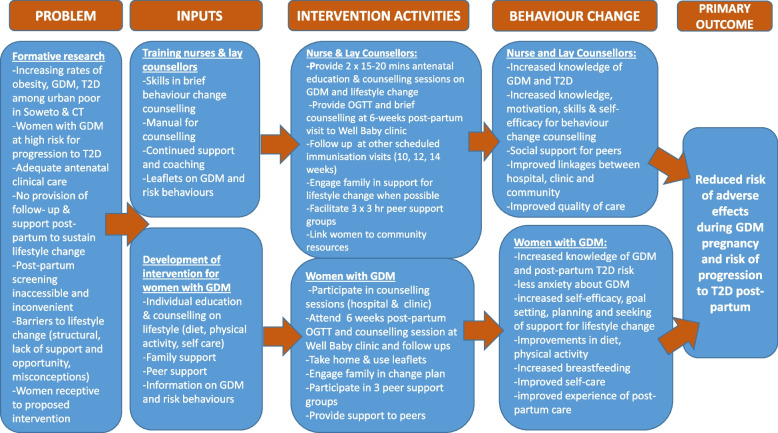
Logic model for the IINDIAGO intervention

The logic model in Fig. 3 provides an overview of the prototype IINDIAGO intervention: its inputs, activities, proposed mechanisms for change and expected outcomes. The intervention targeted both women with GDM and healthcare providers for change. Our consultations with the Department of Health suggested that it was most feasible and appropriate to give lay counsellors the central role in delivering the intervention. Lay counsellors are principally employed in the SA public health system for HIV education and counselling, but the scope of their work is increasingly being expanded to include NCD prevention and management. They are drawn from the local community and are valued for their intimate knowledge of the circumstances, language, and cultural values of the local population. We called our counsellors Healthy Living Coaches (HLCs) to emphasise their supportive role.

In our intervention plan, their responsibilities included: educating and counselling women attending the GDM clinic at the tertiary level of antenatal care; offering the 6 week post-partum OGTT test during the mother’s first visit to the Well Baby clinic for immunisation; providing continued diabetes education, behaviour change counselling and social support (in the same setting at every scheduled immunisation visit: 6, 10, 12, 14, 36 weeks); organising three peer support groups in the community; and making home visits if women wanted help in educating the family. We planned for an experienced diabetes nurse to undertake the initial counselling in the antenatal clinic and to play an important role in supporting and supervising the lay counsellors. Both the nurse and lay counsellors were to be trained in a patient-centred, motivational approach to behaviour change counselling, drawing on the principles and methods of Motivational Interviewing [44] and Healthy Conversations [45] (see Supplementary file 4 for outline of training).

These approaches challenge the traditional prescriptive, advice-giving approach by suggesting that the role of the counsellor is to facilitate consideration of behaviour change by eliciting arguments for change from the patient/client themselves and by helping them articulate and resolve their feelings of ambivalence about change. A key characteristic is an empathetic therapeutic style which emphasises the importance of active listening in order to understand the client’s perspective and respecting their autonomy in decision making [46]. From our qualitative research with women [37, 41], it was clear that they were deeply dissatisfied with the current approach which simply dictated what they should do and how to do it, with little opportunity for engagement and consideration of their needs and concerns. They expressed a strong need for a more collaborative education and counselling style, which included them as active participants and gave them opportunities to ask questions, express their feelings and supported them in making informed choices about lifestyle change that were realistic in their circumstances and congruent with their cultural/family traditions. The facilitated peer group sessions were planned as an opportunity for women to draw on each other for social support, peer-based problem solving, transfer of skills and modelling. The plans for each of these sessions are outlined in Table 3 below.

**Table 3:** Outline of peer support group sessions

Overall objectives:
1. To connect women with counsellor and with other women in the intervention cluster 2. To provide community-based peer support in line with the key messages of the intervention programme 3. Demonstrate practical activities which bridge theory into practice 4. To create a non-clinical ‘safe space’ for women to express their concerns, ask questions and share experiences 5. To welcome an opportunity for partners, family members or friends to participate in the intervention
Healthy Living Coach (HLC) to make all practical arrangements for the community-based support group sessions: book/arrange suitable venue, ensure minimum number of participants at set time, organise refreshments, keep attendance register and records for process evaluation, invite experts if needed/wanted HLC are provided with a general format for each session, including activities and materials relevant to each theme **General Format:** 1. Icebreaker 2. Introduction from HLC about the theme of each session and why it is important to attend all three sessions 3. What does x mean to you (being healthy, eating well, physical activity, self-care)? HLC to elicit baseline of knowledge and build on what they know 4. Short presentation on Diet/ Physical Activity/Self-care 5. Group activities: eg cooking demonstrations, physical activity, group sharing, meditation 6. Discussion and feed back
**GROUP 1: Making Healthier Choices (CAPABILITY)—The WHAT**
Objectives: 1. To develop the knowledge and skills to enable behaviour change in diet, physical activity and self-care 2. To demonstrate skills through practical activities
**GROUP 2: Finding Solutions (OPPORTUNITY)—The HOW**
Objectives: 1. To share ideas and experiences for how to overcome common barriers in the social and physical environment 2. To generate context-specific and realistic solutions in a supportive group setting
**GROUP 3: Self-care and becoming an agent of change (MOTIVATION)—The WHY**
Objectives: 1. To develop a sense of agency to change oneself and one’s family by sharing success stories and practical strategies 2. To align healthy lifestyle behaviours with core values and identity 3. To raise greater self-awareness of mental and physical health and convey a more holistic approach to health 4. To reinforce the concept of women becoming their own “body experts”—informed and proactive patients

Case records were developed to assist the lay counsellors keep meticulous records for process evaluation purposes and to guide them through the key tasks of each session. Regular group feedback meetings were planned to give them opportunities to support each other and discuss how to address problems. Ongoing coaching and role play practice were planned for the first few months of the intervention, building on and reinforcing the initial training in counselling.

## Discussion

In this paper we have illustrated how we used the BCW framework and COM-B model to design an intervention to assist women with GDM to reduce their risk of adverse effects during pregnancy and prevent progression to T2D.

The intervention is currently being tested against usual care in an exploratory trial – as recommended by the MRC Framework for the Development of Complex Interventions (the protocol for the trial is being reported elsewhere). Following the results of this study, modifications are likely to be made to the intervention and study design in preparation for a full clinical trial to assess effectiveness.

In our experience, although exacting and time consuming, the BCW was a valuable tool to use in designing our intervention and tailoring its content and format to our target population and local context. Following the BCW process prompted a rigorous analysis of the problem and how it could potentially be addressed in our setting. It allowed for the consideration and incorporation of evidence from various sources: the research literature, the findings from our primary formative research with GDM women and stakeholders (both qualitative and quantitative), as well as the judgements of the research team and other stakeholders, based on their accumulated knowledge and experience. It guided us through a systematic, step-by-step process, moving from identifying and selecting the relevant target behaviours, to a thorough analysis of these behaviours and the barriers to change according to the COM-B model, to identifying the most promising, feasible and culturally appropriate behaviour change techniques, intervention functions and modes of delivery in our context. Using this model provided a robust and transparent theoretical foundation on which to develop our intervention, assisted us in making explicit the hypothesised pathways for behaviour change and enabled us to describe the intervention in standardised, precisely defined terms.

Other intervention studies have reported similar benefits from using the BCW and COM-B model. These include other reports on the development of interventions aimed at improving physical activity and/or diet [47–49] and smoking cessation among indigenous Australian pregnant women [50]. To our knowledge, there are only a handful of studies which describe the use of BCW in the development of interventions specifically for GDM women. The STAR MAMA project [51] concluded that using the BCW enabled a much more thorough and detailed planning process for the development of their intervention, which used an automated, telephone call system to deliver tailored diabetes prevention messaging to Latino women with prior GDM. The Stay-Active project [52], which developed a smart phone app to increase physical activity among GDM women attending NHS services, found, as we did, that mapping their findings from their focus groups with GDM women onto the COM-B model and TDF helped them focus on what needed to change for the target behaviour to occur, to address specific barriers to and enablers for behaviour change in their context, and then to link these to appropriate behaviour change techniques. Flannery et al. (2018) [53] similarly highlight the value of the COM-B and TDF in making a diagnosis of the barriers and facilitators to physical activity as a basis on which to develop an intervention for at risk, obese pregnant women.

There is broad consensus in these reports that the BCW is an accessible, comprehensive and flexible methodology for intervention design and that the stepped approach assists developers to conduct a rigorous behavioural diagnosis that captures the complexity of health behaviour, link theory to intervention content and strategy, and consider a range of intervention functions and policy options. Further, the explicit account of the development process and the assumptions about the intervention required by the BCW facilitates the subsequent evaluation of the operationalisation of the selected behaviour change techniques and process evaluation [47–49, 54]. However, there are also some notable caveats in using the BCW framework: firstly, it requires considerable time. In our case, the programme of formative research took about 2 years, and the iterative process of input and design took over a year, along with preparing for the trial (others have reported between 12 -24 months). Furthermore, for a good outcome, the application of the BCW requires the skills of health professionals from multiple disciplines and the leadership of someone with a thorough understanding of the model, preferably a behavioural scientist. In our experience, the amount of time and the in-depth knowledge required of the formative data, the COM-B model, TDF domains and the BCTs for the behavioural analysis and mapping steps, made it difficult to consistently involve other members of the research team or stakeholders in this part of the BCW process, limiting their input to the workshop and various feedback meetings. Similarly, this may be a limitation to the participation of GDM women or healthcare providers in a co-production type of design process. Maindal et al. (2021) who developed a similar intervention for GDM women in Denmark using the Hawkins’ co-production (2017) framework, rather than the BCW, report that their evidence-based, iterative design process, which involved multiple stakeholders, was immensely complex, and time and resource consuming [55, 56]. Reflecting on this, they argue that is important that future intervention studies closely monitor and cost the resources required, but at the same time, they conclude that the thoroughness of the approach was essential for creating intervention ownership, relevance, and effectiveness.

A further limitation of the BCW is that while there is some evidence for the acceptability, validity, and reliability of self-evaluating COM-B, there is no standard measure with which to test the predictive validity of COM-B or to assess the impact of interventions based on COM-B model [57–59].

Criticism has also been levelled at the COM-B model by Marks [60], who argues that the model’s omission of 'Wanting’ as a crucial causal factor in behaviour change, makes it unfit for purpose ‘Wanting’ is defined as the mental state that motivates an individual or group towards a goal that is desirable, but not essential for survival. Whilst raising questions of the models’ explanation of how the various determinants interact to cause behaviour change may well be warranted, we understood Motivation in the COM-B model to include the element of both ‘need’ and ‘want’. In the TDF, the domain of Motivation includes aspects such as identity, beliefs, optimism and emotion which build motivation, desire and need. The BCW manual also suggests that interview questions for this domain include ones which explore how much and why a person may or may not want to do X.

## Conclusion

This paper offers an in-depth description of designing a complex intervention tailored to the challenging contexts of urban South Africa and embedded in the existing health system. To date, much of the reporting on behaviour change interventions has lacked adequate detail on the process of development, intervention content, definition of behaviour change techniques and an underlying theoretical rationale. This makes it difficult for the results to be judged reproducible or to draw conclusions about intervention effects and causal mechanisms. This limitation can be partly attributable to the fact that interventions are commonly designed without the use of evidence-based models or behaviour change theory [61–64]. Our paper contributes to addressing this lack in the literature by describing the intervention development process in sufficient detail to allow for greater insight into the active ingredients of our intervention, improved interpretation of the findings of the RCT, replication of the design process in a different setting and for comparisons to similar interventions.

Use of such a systematic and analytical approach is likely to optimise the feasibility and efficacy of interventions, as well as the quality of evaluation. If such rigour is to become a standard of good practice, research agencies and funders need to recognise the need to allocate sufficient personnel, time and funds for this purpose and regard it as a worthwhile investment in enhancing the prospect of producing effective interventions.

## Supplementary Information

**Additional file 1.** IINDIAGO matrix: Expanded COM-B: TDF domains, theoretical constructs and relevance to GDM women (identified barriers & enablers): Physical Activity.

**Additional file 2.** IINDIAGO matrix: Expanded COM-B: TDF domains, theoretical constructs and relevance to GDM women (identified barriers & enablers): Self-care.

**Additional file 3.** Resources for IINDIAGO intervention.

**Additional file 4.** Outline of training for lay counsellors.

## Data Availability

The development of the intervention drew on data generated through a programme of formative research (see Table 1 on page 10). The datasets for each of these studies is available from the relevant lead researcher/first author upon reasonable request. The references for each published paper are presented in Table 1. These have the contact details for each of the corresponding authors.

## References

[1] American Diabetes A: 2 (2017). Classification and Diagnosis of Diabetes. Diabetes Care.

[2] Ye W, Luo C, Huang J, Li C, Liu Z, Liu F. Gestational diabetes mellitus and adverse pregnancy outcomes: systematic review and meta-analysis. BMJ. 2022;377:e67946. 10.1136/bmj-2021-067946. 10.1136/bmj-2021-067946 PMC9131781 35613728

[3] Murphy HR, Howgate C, O’Keefe J, Myers J, Morgan M, Coleman MA, Jolly M, Valabhji J, Scott EM, Knighton P. Characteristics and outcomes of pregnant women with type 1 or type 2 diabetes: a 5-year national population-based cohort study. Lancet Diabetes Endocrinol. 2021;9(3):153–64. 10.1016/S2213-8587(20)30406-X 33516295

[4] Zhang C, Olsen SF, Hinkle SN, Gore-Langton RE, Vaag A, Grunnet LG, Yeung EH, Bao W, Bowers K, Liu A (2019). Diabetes & Women’s Health (DWH) Study: An observational study of long-term health consequences of gestational diabetes, their determinants and underlying mechanisms in the USA and Denmark. BMJ Open.

[5] Kramer CK, Campbell S, Retnakaran R (2019). Gestational diabetes and the risk of cardiovascular disease in women: a systematic review and meta-analysis. Diabetologia.

[6] Kim SY, England JL, Sharma JA, Njoroge T. Gestational diabetes mellitus and risk of childhood overweight and obesity in offspring: a systematic review. Exp Diab Res. 2011;2011.541308. 10.1155/2011/541308. 10.1155/2011/541308 PMC3179897 21960991

[7] Wang J, Wang L, Liu H, Zhang S, Leng J, Li W, Zhang T, Li N, Li W, Baccarelli AA (2018). Maternal gestational diabetes and different indicators of childhood obesity: a large study. Endocr Connect.

[8] Chivese T, Norris SA, Levitt NS (2019). Progression to type 2 diabetes mellitus and associated risk factors after hyperglycemia first detected in pregnancy: A cross-sectional study in Cape Town, South Africa. PLoS Med.

[9] Macaulay S, Ngobeni M, Dunger DB, Norris SA (2018). The prevalence of gestational diabetes mellitus amongst black South African women is a public health concern. Diabetes Res Clin Pract.

[10] Shisana O, Labadarios D, Rehle T, Simbayi L, Zuma K, Dhansay A, Reddy P, Parker W, Hoosain E, Naidoo P (2014). The South African National Health and Nutrition Examination Survey, 2012: SANHANES-1: the health and nutritional status of the nation.

[11] Martis R, Crowther CA, Shepherd E, Alsweiler J, Downie MR, Brown J. Treatments for women with gestational diabetes mellitus: an overview of Cochrane systematic reviews. Cochrane Db Syst Rev. 2018;8(8):CD012327. 10.1002/14651858.CD012327.pub2. 10.1002/14651858.CD012327.pub2 PMC6513179 30103263

[12] Rasmussen L, Poulsen CW, Kampmann U, Smedegaard SB, Ovesen PG, Fuglsang J (2020). Diet and healthy lifestyle in the management of gestational diabetes mellitus. Nutrients.

[13] Guo J, Chen JL, Whittemore R, Whitaker E (2016). Postpartum Lifestyle Interventions to Prevent Type 2 Diabetes Among Women with History of Gestational Diabetes: A Systematic Review of Randomized Clinical Trials. J Womens Health (Larchmt).

[14] Brown J, Alwan NA, West J, Brown S, McKinlay CJ, Farrar D, Crowther CA. Lifestyle interventions for the treatment of women with gestational diabetes. Cochrane Db Syst Rev. 2017;5(5):CD011970. 10.1002/14651858.CD011970.pub2. 10.1002/14651858.CD011970.pub2 PMC6481373 28472859

[15] Toi PL, Anothaisintawee T, Chaikledkaew U, Briones JR, Reutrakul S, Thakkinstian A (2020). Preventive role of diet interventions and dietary factors in type 2 diabetes mellitus: an umbrella review. Nutrients.

[16] OuYang H, Chen B, Abdulrahman A-M, Li L, Wu N. Associations between gestational diabetes and anxiety or depression: a systematic review. J Diab Res. 2021;2021:9959779. 10.1155/2021/9959779. 10.1155/2021/9959779 PMC8337159 34368368

[17] Egan AM, Dunne FP, Lydon K, Conneely S, Sarma K, McGuire BE (2017). Diabetes in pregnancy: worse medical outcomes in type 1 diabetes but worse psychological outcomes in gestational diabetes. QJM.

[18] Gilbert L, Gross J, Lanzi S, Quansah DY, Puder J, Horsch A (2019). How diet, physical activity and psychosocial well-being interact in women with gestational diabetes mellitus: an integrative review. BMC Pregnancy Childbirth.

[19] Harrison CL, Brown WJ, Hayman M, Moran LJ, Redman LM. The role of physical activity in preconception, pregnancy and postpartum health. In: Seminars in reproductive medicine: 2016: Thieme Medical Publishers. 2016;34(2):e28-37. 10.1055/s-0036-1583530. 10.1055/s-0036-1583530 PMC6986386 27169984

[20] Koh D, Miller YD, Marshall AL, Brown WJ, McIntyre D (2010). Health-enhancing physical activity behaviour and related factors in postpartum women with recent gestational diabetes mellitus. J Sci Med Sport.

[21] Wing RR, Goldstein MG, Acton KJ, Birch LL, Jakicic JM, Sallis JF, Smith-West D, Jeffery RW, Surwit RS (2001). Behavioral science research in diabetes: lifestyle changes related to obesity, eating behavior, and physical activity. Diabetes Care.

[22] Hod M, Kapur A, Sacks DA, Hadar E, Agarwal M, Di Renzo GC, Roura LC, McIntyre HD, Morris JL, Divakar H (2015). The International Federation of Gynecology and Obstetrics (FIGO) Initiative on gestational diabetes mellitus: A pragmatic guide for diagnosis, management, and care#. Int J Gynecol Obstet.

[23] Khangura S (2010). What is known about postpartum intervention for women with a history of GDM? Knowledge to Action Evidence Summary.

[24] Peacock AS, Bogossian F, McIntyre HD, Wilkinson S (2014). A review of interventions to prevent Type 2 Diabetes after Gestational Diabetes. Women Birth.

[25] HedeagerMomsen AM, Høtoft D, Ørtenblad L, FriisLauszus F, Krogh RHA, Lynggaard V, Juel Christiansen J, TerkildsenMaindal H, Vinther Nielsen C (2021). Diabetes prevention interventions for women after gestational diabetes mellitus: an overview of reviews. Endocrinol Diab Metabol.

[26] Kragelund Nielsen K, GrothGrunnet L, TerkildsenMaindal H, Workshop DDA, Speakers W (2018). Prevention of Type 2 diabetes after gestational diabetes directed at the family context: a narrative review from the Danish Diabetes Academy symposium. Diabet Med.

[27] Tewari A, Praveen D, Madhira P, Josyula LK, Joshi R, Kokku SB, Garg V, Rawal I, Chopra K, Chakma N. Feasibility of a lifestyle intervention program for prevention of diabetes among women with prior gestational diabetes mellitus (LIVING study) in South Asia: a formative research study. Frontiers in Global Women’s Health. 2020;1:587607. 10.3389/fgwh.2020.587607 PMC8594035 34816163

[28] Dennison R, Ward R, Griffin S, Usher-Smith J. Women’s views on lifestyle changes to reduce the risk of developing type 2 diabetes after gestational diabetes: a systematic review, qualitative synthesis and recommendations for practice. Diabet Med. 2019;36(6):702–17. 10.1111/dme.13926 PMC6563496 30723968

[29] Fonge YN, Jain VD, Harrison C, Brooks M, Sciscione AC (2020). Examining the relationship between food environment and gestational diabetes. Am J Obstet Gynecol MFM.

[30] Porter AK, Rodríguez DA, Frizzelle BG, Evenson KR (2019). The association between neighborhood environments and physical activity from pregnancy to postpartum: a prospective cohort study. J Urban Health.

[31] Lie M, Hayes L, Lewis-Barned N, May C, White M, Bell R. Preventing type 2 diabetes after gestational diabetes: women’s experiences and implications for diabetes prevention interventions. Diabet Med. 2013;30(8):986–93. 10.1111/dme.12206 23534548

[32] Li N, Yang Y, Cui D, Li C, Ma RC, Li J, Yang X (2021). Effects of lifestyle intervention on long-term risk of diabetes in women with prior gestational diabetes: A systematic review and meta-analysis of randomized controlled trials. Obes Rev.

[33] Craig P, Dieppe P, Macintyre S, Michie S, Nazareth I, Petticrew M (2008). Medical Research Council G: Developing and evaluating complex interventions: the new Medical Research Council guidance. BMJ.

[34] Michie S, Atkins L, West R (2014). The behavior change wheel: a guide to designing interventions.

[35] Croot L, O’Cathain A, Sworn K, Yardley L, Turner K, Duncan E, Hoddinott P (2019). Developing interventions to improve health: a systematic mapping review of international practice between 2015 and 2016. Pilot Feasibility Stud.

[36] Muhwava LS, Murphy K, Zarowsky C, Levitt N. Policies and clinical practices relating to the management of gestational diabetes mellitus in the public health sector, South Africa – a qualitative study. BMC Health Serv Res. 2018;18(1):349. 10.1186/s12913-018-3175-x. 10.1186/s12913-018-3175-x PMC5946476 29747657

[37] Muhwava LS, Murphy K, Zarowsky C, Levitt N (2019). Experiences of lifestyle change among women with gestational diabetes mellitus (GDM): A behavioural diagnosis using the COM-B model in a low-income setting. PLoS ONE.

[38] Krige SM, Booley S, Levitt NS, Chivese T, Murphy K, Harbron J (2018). Dietary intake and beliefs of pregnant women with gestational diabetes in Cape Town, South Africa. Nutrients.

[39] Mutabazi JC, EnokBonong PR, Trottier H, Ware LJ, Norris SA, Murphy K, Levitt N, Zarowsky C (2021). Integrating gestational diabetes and type 2 diabetes care into primary health care: Lessons from prevention of mother-to-child transmission of HIV in South Africa-A mixed methods study. PLoS ONE.

[40] Mutabazi JC, Gray C, Muhwava L, Trottier H, Ware LJ, Norris S, Murphy K, Levitt N, Zarowsky C (2020). Integrating the prevention of mother-to-child transmission of HIV into primary healthcare services after AIDS denialism in South Africa: perspectives of experts and health care workers-a qualitative study. BMC Health Serv Res.

[41] Muhwava LS, Murphy K, Zarowsky C, Levitt N. Perspectives on the psychological and emotional burden of having gestational diabetes amongst low-income women in Cape Town. South Africa BMC Women’s Health. 2020;20(1):1–12. 10.1186/s12905-020-01093-4 PMC7552378 33046050

[42] Everett-Murphy K, Pentecost M, Muhwava L, Majikela-Dlangamandla B (2017). The Experience of Conducting Focus Group Discussions on the Topic of Gestational Diabetes in Cape Town.

[43] Khangura SGJ, Moher D (2010). What is known about postpartum intervention for women with gestational diabetes mellitus? Ottawa Hospital Research Institute.

[44] Rollnick S, Miller WR (1995). What is motivational interviewing?. Behav Cogn Psychother.

[45] Lawrence W, Cheminade C, Rahman E, Hankinson A (2018). Making Every Contact Count Programme Training Manual. Health Education England.

[46] Emmons KM, Rollnick S (2001). Motivational interviewing in health care settings: opportunities and limitations. Am J Prev Med.

[47] McEvoy CT, Moore SE, Appleton KM, Cupples ME, Erwin C, Kee F, Prior L, Young IS, McKinley MC, Woodside JV (2018). Development of a peer support intervention to encourage dietary behaviour change towards a Mediterranean diet in adults at high cardiovascular risk. BMC Public Health.

[48] Kinnear F, Wainwright E, Bourne J, Lithander F, Hamilton-Shield J, Searle A (2020). The development of a theory informed behaviour change intervention to improve adherence to dietary and physical activity treatment guidelines in individuals with familial hypercholesterolaemia (FH). BMC Health Serv Res.

[49] Moore AP, Rivas CA, Stanton-Fay S, Harding S, Goff LM (2019). Designing the Healthy Eating and Active Lifestyles for Diabetes (HEAL-D) self-management and support programme for UK African and Caribbean communities: a culturally tailored, complex intervention under-pinned by behaviour change theory. BMC Public Health.

[50] Gould GS, Bar-Zeev Y, Bovill M, Atkins L, Gruppetta M, Clarke MJ, Bonevski B (2017). Designing an implementation intervention with the Behaviour Change Wheel for health provider smoking cessation care for Australian Indigenous pregnant women. Implement Sci.

[51] Handley MA, Harleman E, Gonzalez-Mendez E, Stotland NE, Althavale P, Fisher L, Martinez D, Ko J, Sausjord I, Rios C. Applying the COM-B model to creation of an IT-enabled health coaching and resource linkage program for low-income Latina moms with recent gestational diabetes: the STAR MAMA program. Implementation Sci. 2016;11(1):73. 10.1186/s13012-016-0426-2. 10.1186/s13012-016-0426-2 PMC4870786 27193580

[52] Smith R, Michalopoulou M, Reid H, Riches SP, Wango Y, Kenworthy Y, Roman C, Santos M, Hirst J, Mackillop L (2022). Applying the behaviour change wheel to develop a smartphone application ‘stay-active’to increase physical activity in women with gestational diabetes. BMC Pregnancy Childbirth.

[53] Flannery C, McHugh S, Anaba AE, Clifford E, O’Riordan M, Kenny LC, McAuliffe FM, Kearney PM, Byrne M (2018). Enablers and barriers to physical activity in overweight and obese pregnant women: an analysis informed by the theoretical domains framework and COM-B model. BMC Pregnancy Childbirth.

[54] Moore GF, Audrey S, Barker M, Bond L, Bonell C, Hardeman W, Moore L, O’Cathain A, Tinati T, Wight D, et al. Process evaluation of complex interventions: Medical Research Council guidance. BMJ. 2015;350:h1258. 10.1136/bmj.h1258 PMC4366184 25791983

[55] Maindal HT, Timm A, Dahl-Petersen IK, Davidsen E, Hillersdal L, Jensen NH, Thøgersen M, Jensen DM, Ovesen P, Damm P (2021). Systematically developing a family-based health promotion intervention for women with prior gestational diabetes based on evidence, theory and co-production: the Face-it study. BMC Public Health.

[56] Hawkins J, Madden K, Fletcher A, Midgley L, Grant A, Cox G, Moore L, Campbell R, Murphy S, Bonell C (2017). Development of a framework for the co-production and prototyping of public health interventions. BMC Public Health.

[57] Willmott TJ, Pang B, Rundle-Thiele S (2021). Capability, opportunity, and motivation: An across contexts empirical examination of the COM-B model. BMC Public Health.

[58] Keyworth C, Epton T, Goldthorpe J, Calam R, Armitage CJ (2020). Acceptability, reliability, and validity of a brief measure of capabilities, opportunities, and motivations (“COM-B”). Br J Health Psychol.

[59] Howlett N, Schulz J, Trivedi D, Troop N, Chater A (2019). A prospective study exploring the construct and predictive validity of the COM-B model for physical activity. J Health Psychol.

[60] Marks DF (2020). The COM-B system of behaviour change: Properties, problems and prospects. Qeios.

[61] Prestwich A, Sniehotta FF, Whittington C, Dombrowski SU, Rogers L, Michie S (2014). Does theory influence the effectiveness of health behavior interventions? Meta-analysis. Health Psychol.

[62] Herber OR, Atkins L, Störk S, Wilm S (2018). Enhancing self-care adherence in patients with heart failure: a study protocol for developing a theory-based behaviour change intervention using the COM-B behaviour model (ACHIEVE study). BMJ Open.

[63] Michie S, Johnston M, Francis J, Hardeman W, Eccles M (2008). From Theory to Intervention: Mapping Theoretically Derived Behavioural Determinants to Behaviour Change Techniques. Appl Psychol.

[64] Michie S, van Stralen MM, West R (2011). The behaviour change wheel: a new method for characterising and designing behaviour change interventions. Implement Sci.

