# Supplementary material for: Using the COM-B model and Behaviour Change Wheel to develop a theory and evidence-based intervention for women with gestational diabetes (IINDIAGO)
Source: BMC Public Health. 2023 May 15;23:894. doi: 10.1186/s12889-023-15586-y (PMC10186807; doi:10.1186/s12889-023-15586-y)
Supplement: Supplementary file 1 — Additional file 1. IINDIAGO matrix: Expanded COM-B: TDF domains, theoretical constructs and relevance to GDM women (identified barriers & enablers): Physical Activity. [file 12889_2023_15586_MOESM1_ESM.docx]

| **Capability**  ***Psychological*** | **Formative Assessment** | **For BC to occur GDM women would need to** | **INT Function** | **BCTS** | **INT Activities and Resources** |
| --- | --- | --- | --- | --- | --- |
| **Knowledge**  An awareness of the existence of something  *Do you know about x?* | - They do know that PA is beneficial to health, but received no info or messaging about PA and GDM (emphasis is on diet) - Do not have materials/ resources on PA to take home | - Receive info on PA during GDM pregnancy and the role in preventing T2D - Need information about what constitutes PA vs. exercise or sport - Understand how PA impacts on glucose levels | **EDUCATION**   - Provide consistent standardized, appealing and simple info and identify several key messages for counselling - Provide resources they can take home - Provide evidence based recommendations for PA for GDM women, adults in general, and children. | 5.1  Info abut health consequences | - Present a clear definition of PA in counseling and peer group - IC4H PA brochure and diabetes leaflet provide info on health consequences - Do’s and don’ts for PA for GDM women specifically - Individual counselling tailored to what they already know - Heart and Stroke Foundation leaflet and DVD on PA and pregnancy |
| **Cognitive, interpersonal skills**  *Do you know how to do x?* | - Lack interpersonal skills to challenge social and family social norms about PA | - Gain communication skills to persuade family to become more physically active, less sedentary | **TRAINING**   - Need opportunity to practice communication skills and share experiences about how to be effective change agents in their family | 15.2  Mental rehearsal of successful performance  4.1  Instruction on how to perform the behaviour | - In peer group – ‘Agent for Change’ provide opportunities for problem solving and devising strategies for negotiating change - Home visits -CHW support woman in discussing and negotiating change in the family lifestyle |
| **Memory, Decision, process and attention**  *Is x something you usually do?* | - Minimal focus on PA as part of everyday life. - Many women have not made any decisions to increase PA levels | - Pay greater attention to PA and making choices around increasing levels of PA for long term (during and after pregnancy) - To apply their knowledge about PA to making personal choices that will be feasible and realistic for them | **ENABLEMENT**   - Assist women in identifying opportunities and strategies to incorporate PA into their daily lives - Provide social support and feedback   **TRAINING**   - Provide demonstrations of PA in everyday situation and setting | 8.2  Behaviour substitution  3.2  Social support  6.1  Demonstration of behaviour | - IC4H brochure On PA pg 10 - Brainstorming ideas in the Healthy Choices peer workshop - Chair exercise in Healthy Choices peer workshop |
| **Behaviour regulation**  *Do you have systems or tools for monitoring whether you have carried out x?* | - Don’t have a tool to assess level of PA against recommendations - PA not emphasised enough as a way to regulate glucose and weight gain in existing health services - Not assessed for levels of PA | - Need tools/systems in place that support them to self-monitor their PA against recommendations and their personal fitness goals | **TRAINING**   - Provide means to enable them to assess level of physical activity against recommendations - Offer PA strategies for glucose regulation | 2.3  Self-monitoring of behaviour  2.4  Self-monitoring of outcome  2.6  Biofeedback  1.5  Review of behavioural goals | - p7 IC4H brochure assessment tool - p15 keeping track of PA in brochure - R2H card to help self-monitoring and as a tool for HCP to discuss progress - Use Health Diary which records how doing PA makes them feel - HCPs to jointly review progress with PA from baseline - Referral to smart phone apps to track fitness |

| **Capability**  ***Physical*** | **Formative Assessment** | | **For BC to occur GDM Women would need to** | | **INT Function** | | **BCTS** | | **INT Activities and Resources** | |
| --- | --- | --- | --- | --- | --- | --- | --- | --- | --- | --- |
| **Physical Skills**  Do you have physical skills to perform x? | - GDM women have certain physical limitations given their pregnant state and diabetes related complications | | NA | | N/A | | N/A | | N/A | |
| **Motivation**  *Reflective* | **Formative Assessment** | | **For BC to occur GDM Women would need to** | | **INT Function** | | **BCTS** | | **INT Activities and Resources** | |
| **Social role and Identity**  Is doing x compatible with identity | - Identity as a pregnant woman not associated with doing PA - Prioritise their role as mothers and carers, but don’t fully recognise their potential as role models for PA in their family - Many women regard being voluptuous/curvy as part of a positive cultural/African identity - Having large children is also regarded in a positive light indicating wellness, ability to provide, attractiveness - Do not identify as “sporty” | | - Expand current maternal identity to include caring about NCD prevention in their families through increased PA. - Identify with a physically active lifestyle during and after pregnancy - Consider the potential health risks of the existing cultural norms around acceptable weight | | **PERSUASION**   - Affirm role as mothers and as carers of family while expanding it to include NCD prevention - Emphasis role of mother as change agent in family and potential role model for PA for their kids | | 5.2  Salience of health consequences  13.1  Identification of self as a role model  13.4  Valued self-identity  13.2  Reframing  13.5  Identity associated with changed behaviour | | - In the peer group ‘Agent for Change’ do values exercise – advise person to reflect on personal strengths and values before advocating change. - Affirm identity as a physically active person - Use body image scale to discuss healthy body weight and cultural beliefs | |
| **Beliefs above capabilities**  *How difficult/ easy is it to do x?* | - Believe that being overweight and pregnant makes it very difficult to do PA - Believe that living in poor circumstances makes it difficult to do PA (safety, lack of facilities, safe open, green space, crowded roads, lack of money for gym or classes) - Believe they don’t have time to do PA | | - Feel more confident that PA during a GDM pregnancy and when overweight is possible - Feel more confident that doing PA is possible even within constraints of their circumstances | | **PERSUASION**   - Enhance self-efficacy to do PA during pregnancy and when overweight - Encourage women that they are capable of PA no matter what size they are - Encourage women to believe that PA is possible, even given constraints of circumstances   **ENABLEMENT**   - Assist women in problem solving to overcome barriers   **MODELLING**   - Present real life examples of people in similar circumstances finding ways of doing PA | | 15.1 Verbal persuasion about capability  3.1Social support  1.2Problem solving  6.1 Demonstration of behaviour. | | - Individual counselling- using a non-directive approach to enhance self-efficacy and self-autonomy - Peer group “overcoming barriers” - Collective problem solving overcoming shared obstacle can enhance belief that it is possible despite circumstances - Use “health at every size” approach which emphasises that improving fitness through increased physical activity does not necessarily dictate losing weight as a goal i.e. possible to become physically fitter even if weight stays the same - IC4H brochure testimonials - MI confidence scale to elicit difficulties and facilitation | |
| **Optimism**  *How confident are you that the problems of implementing x can be solved?* | | - Women tend to believe that T2D is inevitable if there is history in the family —fatalistic | | - Feel optimistic that they can avoid progression to T2D through a healthy lifestyle which includes PA even if there is family history - Aspire to be like others who have achieved PA | | **ENABLEMENT**   - Emphasise their agency in preventing T2D   **PERSUASION**   - Dispel Beliefs about T2D being inevitable if it exists in family history   **MODELLING**   - Provide examples of people like them who have achieved PA | | 15.1  Verbal Persuasion about capability  6.2 modelling | | - Non directive counseling approach builds optimism and self-efficacy - Testimonials in IC4H brochure to model behaviour - Peer group “Overcoming Barriers” -sharing of success stories in person |
| **Beliefs about consequences**  What do you think will happen if you do x? | | - Underestimate the effects of physical inactivity on health and well-being - Believe that consequences of GDM relate to self only and only during pregnancy - Believe PA during a GDM pregnancy is potentially harmful to baby and could experience pain, bring on pre-mature labour or miscarriage | | - Assess risk more accurately - Make a clearer link between GDM and future risk - Believe in benefits of PA for self and family - Believe that PA is possible during a GDM pregnancy without causing harm | | **EDUCATION**   - Explore beliefs and attitudes related to physical activity during pregnancy - Provide tools to assess risk to self and family, which can be shared within family   **PERSUASION**   - Reframe what GDM signifies for self and family - Emphasis PA’s role in NCD prevention - Provide expert information on safe PA during GDM pregnancy | | 13.2  Reframing  5.1  Information about health consequences  9.1  Credible source | | - Peer group “Overcoming Barriers” - Dispel myth related to risks - p11 of IC4H brochure to assess risk - Message from specialist (Dinky) via WhatsApp group or skype |
| **Intentions**  *Have you made a decision to do x?* | | - Majority form intention fo dietary change during pregnancy but not of PA - There is less emphasis on PA in antenatal care | | - Form long term intentions to do regular PA as part of a healthy lifestyle to prevent NCDs | | **ENABLEMENT**   - Encourage and support women in formulating intentions to include PA as part of a healthy lifestyle - assists in decisional balancing | | 9.2  Pros and Cons  9.3  Comparative imagining of future outcomes | | - Readiness to change scale to be used in individual counseling - decisional balance exercise ( integrated with diet) to be used in peer group ‘overcoming barriers’ |
| **Goals**  *How much do you want to do x?*  *What exactly are you going to do?* | | - In general women do not set goals for PA - Lack of support and guidance in realist goal setting | | - Set goals for PA and cutting down on sedentary time - Goals need to personalized and SMART | | **ENABLEMENT**   - Provide support and guidance in realistic SMART goal setting - Affirm small interim goals and successes - Prompt commitment to change for the long term | | 1.1 Goal setting (behaviour)  1.3 Goal setting (outcomes)  1.4 Action planning  8.7 Graded tasks | | - IC4H card - Regular individual counseling to develop SMART goals and to acknowledge/affirm small successes - Referral to Smart phone apps for setting PA goals |
| **Motivation**  *automatic* | | **Formative Assessment** | | **For BC to occur GDM Women would need to** | | **INT Function** | | **BCTS** | | **INT Activities and Resources** |
| **Reinforcement**  *Are there any incentive to doing x?* | | - Sedentary habits are well entrenched as part of everyday life | | - Develop habits involving PA and limiting sedentary time - Start associating people/things/times/environments/contexts with PA | | **TRAINING**   - Provide prompts and tips to aid positive habit formation and reversal of sedentary habits | | 8.3  Habit formation  8.4 Habit reversal | | - Peer group to discuss options for everyday contexts where one might increase PA - IC4H leaflet p 10-11 |
| **Emotions**  *Does doing x evoke and emotional response?* | | - Experience negative emotions around the idea of PA especially during pregnancy ( nausea, fatigue, anxiety about effect on baby) - Shame or embarrassment about doing PA in public if overweight - Negative body image can limit PA | | - Develop positive emotions about doing PA - Recognize relationship between PA and mood/emotional state | | **ENABLEMENT**   - Provide a safe non-judgmental environment for exploring emotions around body image - Provide opportunities for women to do PA and evaluate their emotional states afterwards | | 3.3 Social support (emotional)  5.4 Monitoring of emotional consequences | | - Peer group motivation in ‘agent for change session’ women can do a short physical exercise and evaluate their emotional state afterwards - Activity around body image - being the body expert - journaling activity |
| **Opportunity**  *Enviro and Social* | | **Formative Assessment** | | **For BC to occur GDM Women would need to** | | **INT Function** | | **BCTS** | | **INT Activities and Resources** |
| **Environmental context and resources**  *To what extent do physical/resources factors hinder or facilitate x?* | | - Time is considered a barrier - Few safe and/or aesthetically pleasing environments to do PA - Cost of gyms and transport prevents women from joining - Lack of facilities to PA in local communities | | - Adapt PA to their circumstances - Identify and create forms of PA that are affordable and don’t require a lot of time or special facilitates - Identify feasible opportunities in local environment | | **TRAINING**   - Emphasise that PA can be practiced in any circumstance and incorporated into ordinary daily activities - Provide tips on feasible realist options for PA - Demonstrate desired behaviour - Collect and provide info on local resources | | 6.1 Demonstration of the behaviour  4.1  Instruction on how to perform the behaviour | | Peer group ‘overcoming barriers’  Activity: examples of PA i.e. chair or step exercises  Provide list of local clubs, community centers etc which have classes or spaces for walking or other forms of PA |
| **Social influences**  *To what extent do social influences hinder or facilitate x?* | | - Lack of social support from family and friends - Social norms not conducive to PA in lifestyle - Sedentary behavior and being overweight has become normalized and social accepted - Women more likely to participate in PA if there are organized groups with women near home | | - Feel greater social support - Join groups with other women to exercise - Identify opportunities for PA with children family and friends | | **ENABLEMENT**   - Prompt seeking of social support - Explore and evaluate social norms around PA - Solicit their ideas for forms of PA which can be done alone and in groups - Provide examples/ testimonials that dispel myths about what is socially normal or appropriate | | 3.1  Social support  13.2  Reframing  6.1 Demonstration of the behaviour  6.2  Social comparison | | - Individual counseling to prompt seeking of social support - Peer group ‘Overcoming Barriers’ to discuss identity - Encourage them to find a get fit partner or form local groups - testimonials |
