# Supplementary material for: Using the COM-B model and Behaviour Change Wheel to develop a theory and evidence-based intervention for women with gestational diabetes (IINDIAGO)
Source: BMC Public Health. 2023 May 15;23:894. doi: 10.1186/s12889-023-15586-y (PMC10186807; doi:10.1186/s12889-023-15586-y)
Supplement: Supplementary file 2 — Additional file 2. IINDIAGO matrix: Expanded COM-B: TDF domains, theoretical constructs and relevance to GDM women (identified barriers & enablers): Self-care. [file 12889_2023_15586_MOESM2_ESM.docx]

| **CAPABILTY**  **psychological** | **Formative Assessment** | **For BC to occur GDM women would need to…** | **INT Function**  **What needs to be done to change behaviour** | **BCTS** | **INT activities and Resources** |
| --- | --- | --- | --- | --- | --- |
| **Knowledge**  Do you know about x? | - Many did not fully understand GDM/T2D for themselves and the baby - Not aware of the importance of 6 weeks OGTT and PP g2 monitoring - Do not all receive referral letter for PP test - Not all women know about PP depression and how to differentiate it - Understand stress to encompass different aspects of mental health | - Have basic understanding of GL maintenance and monitoring signs and symptoms - Be aware of the importance of PP GC monitoring/ follow up (Especially 6 week OGTT) - Have basic understanding/Awareness of depression - Info on signs and symptoms of depression, stress and trauma | **EDUCATION**   - Improve health literacy - Provide info on GDM/T2D, how It affects women and baby’s health - Info on managing GL levels (What to do) - Emphasise importance of PP screening - Provide healthy evidence based on targets - Provide info/clear definitions of mental health and stress - Provide messages which link mental and physical wellness (diet, sleep, PA) | 4.1  Instruction on how to perform behaviour  5.1  Info on health consequences (Verbal, visual, written) | - Simple visual aids - Leaflet on GDM and T2D - R2H card provides info on targets - Pregnancy diary - Leaflet on mental wellness. including, pp depression, stress and trauma (PDNSA Perinatal mental health project) |
| **Cognitive and personal skills**  Do you know how to do x? | - Low self-esteem and shyness to ask for support - Under reporting of mental health - Do not know to report stress cognitively | - Need to express how they feel and ask for help and support - Identify signs and symptoms of physical/mental health - Communicate signs and symptoms to health professionals - Know strategies on how to reduce stress | **Training**   - Improve their confidence and ability to communicate health info and mental state - Teach strategies for coping and reducing stress | 8.1 Behavourial practice/rehearsal  4.1  Instruction on how to perform behaviour  11.2  Reduce negative emotions | - Role play - Leaflet on PP depression - Mindfulness practice, relaxation technique, PA, breathing, body scan, meditation in peer group time and management technique |
| **Memory, Attention decision making**  *Is x something you usually do?* | - Don’t pay sufficient attention to their own health, prioritise their families, esp. their babies - Not body aware - Too fatigued or pre-occupied with care of their babies to take care of themselves - Health system focuses more babies than mother PP - FG- conveyed that health systems cared about them - Talk a lot about stress, (financial, motherhood, relationships, unemployment) | - Pay greater attention to their own physical and mental health and well being - Remember to care for themselves PP and keep track of mental and physical sense of well being | ENVIROMENTAL RESTRUCTURING   - Health systems to communicate that it cares about mother’s health PP   **Enablement**   - Focus on own health, as well as the baby’s - Assess woman’s mental health as well as physical health to draw attention to their mental health | 7.1  Prompts and cues  2.2 Feedback on behaviour. | - Follow up PP health system - Referral letter to prompt them to remember to go for OGTT and counselling at 6 weeks - SMS messaging for continued follow up to attend INT. activities - Elicit from women how they feel (open Q’s in counselling; screening tool) to ascertain state of mental health - Self-awareness exercise |
| **Behaviour Regulation**  Anything aimed at managing/changing objective observed or measured actions  *Do you have systems to help you monitor whether you are carrying out x?* | - Don’t have assistance or tools to aid in self-monitoring - System does not engage them in assessing and monitoring own health in PP period | - Play a more active role in self-monitoring and self-management | - Provide a tool to help women identify targets and monitor behaviour change progress - Provide feedback to help monitor changes and progress in self-care | 2.4  Self-monitoring of outcomes  2.2  Feedback on behaviour  2.6  Bio feedback | - R2H Card, 6 week OGTT - Pregnancy diary - Discussion about progress towards goals and individual counselling - Text messaging i.e. thank you for attending your appointment, you are taking good care of yourself. Next appointment is ….. |
| **MOTIVATION**  **Reflective** | **Formative Assessment** | **For BC to occur GDM women would need to…** | **INT Function**  **What needs to be done to change behaviour** | **BCTS** | **INT activities and Resources** |
| **Social role and Identity**  Is doing x compatible with identity? | - Don’t prioritise their own needs self-care, primary identity is care of family/ others - Identity as diabetic normalised in community (high prevalence, considered a family trait) | - See selfcare as being compatible with identity as care - Identity what self-care means for them and strategies that will work for them - Resist normalisation of diabetes in family/community. | - **PERSUASION** - Align identity as mother with self-care - Affirm individual identity and qualities - Enhance self-autonomy and self-determination (‘you have the choice’)   MODELLING   - Provide examples/success stories about mental wellness | 13.2  Re framing  13.5  identity association with behaviour.  15.1  Verbal persuasion about capability  6.1  Demo of behaviour. | - Peer group discussion on strategies for self-care - Exercise: Value Clarification - Mental health leaflets - Testimonial modelling self-care (women who family is diabetic) - See other taking measures to self-care (monitor and manage) in peer group |
| **Beliefs about capabilities**  *How difficult or easy for you to do x?* | - Rely on medical professionals for care especially during pregnancy and medical care - During pregnancy, they don’t receive the kind of counselling that equips/empowers them to actively care for themselves | - Believe that their health is not just the hands of health professionals - Believe that they should actively engage in their own self-care - Become less passive - Enhance self-autonomy | **ENABLEMENT**   - Emphasise that diabetes can be prevented, well managed through their own behaviour. (a diagnosis where patient has a lot of power to control their health   **PERSUASION**   - highlight that HPS only have a limited role | 15.1  Verbal Persuasion about  capability  13.2 Reframing (Pers.) | - Sharing simple, realistic strategies to self-care that they can implement in their context (problem solving with peer group) - In Counselling, use a patient centered empowerment approach - Use Confidence scale - Exercise: rating yourself as own body expert |
| **Optimism**  *How confident are you that the problem of x can be solved?* | - Have fatalistic attitude re diabetes - Live in challenging circumstances which contribute to stress - Ongoing pressure resulting in chronic stress - Low comprehension of GDM causes anxiety - Low self-efficacy affects optimism - GDM seen as additional burden, stressor. | - Feel more confident and optimistic about their ability to self-care - Seek and experience social support | - Enhance self-efficacy - Provide social support (HCP and Peers) - Re assure women GDM can be well Managed/Solved - Prompt seeking of social support from partners and family | 15.1 Verbal Persuasion and capability  15.3 Focus on past success  3.3 Social support  (emotional) | - Mental health leaflet - Peer group support - HCP support and follow up - Share success stories (own or others) - Encourage participation of partners or friend in intervention activities |
| **Beliefs about Consequences**  *What do you think will happen if you do x?* | - Not clear about benefits of self-care - Associated self-care with selfishness, can negatively impact on relationship - Underestimate long term risks signaled by GDM pregnancy | - Believe that taking a more active role in their care will improve outcomes (Physical and mental) - Believe that depression can be effectively treated. - Recognise increased risk of T2D and importance of monitoring PP | **EDUCATION**   - Clarity definition and outcomes of self-care - Provide information on efficiency of treatment for depression   **Persuasion**   - reinforce idea that self-care can positively impact on relationship and make you a better mother | 5.1  Info about consequences  5.6  Info about emotional consequences.  5.2  Salience of consequences | - Exercise showing relationship between diet, PA, Stress, Depression etc. - Info on depression and efficacy of treatment. (Benefits of taking might involve treatment)   (treating depression has effects on body too i.e. sleep, energy etc.   - In counselling, emphasise the risk of T2D to GDM women (+ leaflet) |
| **Intentions**  *Have you made a decision to do x?* | - During pregnancy, much of there is done HCPS - Don’t have long term intentions for self-care after delivery. | - Develop long term intentions for self-care (Inc. Mental and Physical) | - Encourage formulation of long term self-care intentions/decisions - Prompt consideration of future outcomes | 9.2 Pros and cons  9.3 Comparative imagining of future outcomes | - Decisional balances sheet on selfcare - Reflect on results |
| **Goals**  What exactly are you going to do? | - Mental health goals typically not included in self-care for diabetes - HCPS typically sets goals for patients - Passive relationship with HCP has resulted in little personal goal setting. (For physical and mental health) | - Set own goals for self-care - Set achievable goals - Include mental health goals in diabetic action plan - See own goal setting as part of self-care | - Give women support guidance and tools to help them set own realistic goals for self-care inc. their mental health - Prompt planning SMART goals and self-care | 1.4  Action planning  8.7  Graded tasks  1.1  Goal setting  1.3  Goal setting outcome | - Peer group- elicit goal setting for self-care - Exercise – choosing self-care practice for the week ahead - Individual counselling- eliciting goal setting for adherence, (appointment, OGTT, medication) lifestyle change, mental health care - R2HCard |
| **MOTIVATION**  **automatic** | **Formative Assessment** | **For BC to occur GDM women would need to…** | **INT Function**  **What needs to be done to change behaviour** | **BCTS** | **INT activities and Resources** |
| **Reinforcement**  Are there incentives to do x? | - Self-care habits not well established | - Associate self-care practices with specific environment at designated times to aid habit formation through repetition (Taking time yourself, breathing, meditation etc) | **Training**   - **E**licit behaviour strategies for better coping with stress and develop ideas on how to associate them with times and specific environments | 8.3 Habit formation  8.4 Habit reversal | - Home visits – PC does self-care activity with the woman in home environment and gets feedback |
| **Emotion**  Does doing x evoke an emotional response | - Women say the feel better when they self-care - Anxiety because of not fully understanding GDM/T2D not feeling in control of own health | - Women to feel more empowered to take charge of their own health, associate and feelings with self-care practices, - Feel less anxious or stressed about their health management. | **ENABLEMENT**   - Prompt assessment of feelings after performance of self-care activity.   **PERSUASION**   - Provide info about emotional consequences related to self-care/ empowerment. | 5.4 Monitoring of emotional consequences  5.6 Information about emotional consequences | - In peer discussion theme self-care - During home visit – do exercises (e.g. Short meditation) and discuss how they feel afterwards. |
| **OPPORTUNITY**  **environmental** | **Formative Assessment** | **For BC to occur GDM women would need to…** | **INT Function**  **What needs to be done to change behaviour** | **BCTS** | **INT activities and Resources** |
| **Environmental context and resources**  *To what extent do factors in environment or resources help or hinder x?* | - Not aware of local resources for mental health and don’t use them - Women say CHC is too time consuming or inconvenient to attend the 6 week OGTT (long waiting times) - Time, Distance, transport costs prevent them from attending health services for follow up - Lack means ( devices to measure weight etc) to self-monitor health at home | - Know about local which offer mental health services (pp depression; breastfeeding; crisis ; hotlines, etc.) - be able to access the services and intervention services - Need resources to aid them in self-monitoring and self-care | **ENABLEMENT**   - Collate info on local, accessible services specific for each intervention clinic   **ENVIRONMENTAL RESTRUCTURING**   - INT must take place at a convenient accessible, local facility close to them (WB clinic that they are already attending; at their home and at local venue for peer group sessions) | 3.2 Social Support (Practice)  12.2Restructuring the environment  7.1 Prompt and cues | - Resources package foe HCPS and Women - Posters in WBC and venues for peer group - Counselling must include referral to local resources - Counselling in local WB clinics - Peer group at accessible local venue agreed on - Home visits by PC - Referral letter for 6 weeks OGTT and text reminders - Referral to mental health services - PC to have scale, blood pressure cuff, glucose monitoring device(at home or peer group) to aid monitoring and felling in of the R2H card. |
| **OPPORTUNITY**  **social** | **Formative Assessment** | **For BC to occur GDM women would need to…** | **INT Function**  **What needs to be done to change behaviour** | **BCTS** | **INT activities and Resources** |
| **Social influences**  *To what extent do social influences hinder/facilitate doing x?* | - FGs showed women lacked adequate emotional and social support especially PP - Some women who have suffered traumatic pregnancies and deliveries feel isolated deliveries and don’t have opportunities to share their experiences. - They appreciated the opportunity for sharing their experiences/stories in FG and discovered other women had similar experiences - Women generally report stigma surrounding mental health issues and this may prevent them from seeking help | - Participate in peer group discussion on depression and self-care and share experiences - Seek and social support from significant other in their social environment - Involve partners/family members in INT activities - Gain a new perspective on mental health an self-care | **ENABLEMENT**   - Provide opportunities for discussion and support around mental health and self-care - Provide safe environment for sharing experience through competent group facilitation - Involve partners and family in INT to create more social support - Provide a social comparison with others who have experienced mental health issue and got better - Reframe depression as a condition that can be successfully treated | 3.1 Social support (General)  3.3Social Support (Emotional)  6.2Social Comparison  13.2 Reframing. | - Peer group theme “self-care” - Home visits that can engage the family and partner - Info about extent of depression among SA women put into social context (a common problem) - Reassure women that depression can be successfully treated using stats and testimonials - Use PNDSA and perinatal mental health project resources which address stigma |
