# Supplementary material for: Using the COM-B model and Behaviour Change Wheel to develop a theory and evidence-based intervention for women with gestational diabetes (IINDIAGO)
Source: BMC Public Health. 2023 May 15;23:894. doi: 10.1186/s12889-023-15586-y (PMC10186807; doi:10.1186/s12889-023-15586-y)
Supplement: Supplementary file 3 — Additional file 3. Resources for IINDIAGO intervention. [file 12889_2023_15586_MOESM3_ESM.docx]

**Resources for IINDIAGO intervention**

The main resource developed for women in the intervention arm was an IINDIAGO Diary. It contained simple explanations of GDM, its implications for the women herself, her baby and family and the potential for prevention; tailored guidelines for healthy eating and physical activity; and provided space for the woman to record her behaviour change goals. Other resources on diet and physical activity included testimonials with people from the target communities, self-assessment and monitoring tools and tips; for example, on how to read food labels, prepare food in a healthier way and adapt family favourite recipes. Importantly, these resources were only to be offered to women in the context of personalised counselling and not distributed on their own, as this has been found to be a more effective way to use materials (Moore, 2002). See <http://www.ichange4health.co.za/> for the leaflets on diet, physical activity and the recipe books used in the IINDIAGO intervention.

The table below outlines some of the key messages that we planned to incorporate into the resources and counselling sessions.

**S2: Key messages for incorporation into education resources and counselling**

| **Research Finding** | **Key messages to promote optimism and sense of agency** | |
| --- | --- | --- |
| -Women are unaware of long-term risks of GDM  -understand the problem as relevant only to pregnancy | **GDM: an early warning sign of elevated risk for T2D** | - GDM raises a red flag for increased risk for T2D and other NCDs in longer term  - an opportunity to consider making long-term, sustainable changes in lifestyle |
| -Prevention messages not given enough emphasis by health care providers  -women don’t understand *how* eating healthily and being physically active prevents diabetes  -women feel fatalistic about developing T2D | **GDM related harms and T2D can be prevented** | -reassurance that a healthy lifestyle is effective in managing glucose levels and preventing harm to the baby  -progression to T2D can be avoided  -healthy foods and physical activity are ‘the best medicines’  -a healthy lifestyle has psychological benefits too |
| -Women reported that if they had a family history, they expected to get diabetes | **Family history does not mean developing GDM/T2D is inevitable** | -unhealthy lifestyle most important risk factor  -prevalent fatalism is unfounded  -T2D is an avoidable disease |
| -Women feel solely responsible for GDM and behaviour change  -need social support from family to succeed  -feel isolated eating separately from the family | **GDM is not just the woman’s problem** | - raises a red flag for the family as they inevitably share a similar lifestyle  - baby at risk of metabolic disease in early adulthood  - GDM provides a strong rationale for behaviour change in whole family to prevent T2D  -family need to help mother and baby avoid diabetes |
| -Women regard themselves as having the primary role in caring for their families’ health and well being  -women value this role and it is a very important part of their identity | **Being an agent of change in the family, aligns with their valued role as mothers and carers** | -mothers have a vital role to play in protecting the family’s health and well-being  -they need to educate and engage the whole family/household  -peer support can be helpful in this process |
| -Women believe that healthy foods are expensive and don’t ‘fit’ with their culture  - they feel daunted by the challenge  -women have numerous misconceptions of what constitutes healthy foods  -women unaware of the role of large food multinationals in determining our food choices  -women believe that the way past generations grew up eating was better | **Behaviour change is difficult, but not impossible** | -making healthier choices is difficult, but not impossible, even if living in poor socio-economic circumstances  -many people like you have succeeded in adopting healthier lifestyles  -there are ways to eat more healthily within your budget and cultural traditions  -cooking methods and diets can be modified, rather than radically changed  -even small changes can make a big difference  -we live in an obesogenic environment which promotes and reinforces unhealthy choices  -returning to ‘the old way’ of cooking and eating meals at home together is healthier and has other benefits |
| -Women had limited knowledge of how to change their diet in their circumstances | **Knowledge is only the first step in achieving behaviour change** | -knowledge about what to change and how to change is an essential first step  -we also need skills to guide our food choices and prepare healthy food  -problem solving how to overcome common obstacles with our peers can be helpful |
| -women felt that their psychological needs were not sufficiently considered by healthcare providers  -many women experience considerable stress and anxiety related to their circumstances during their GDM pregnancy | **Self-care is important** | -women need to devote time and energy to taking care of themselves  -good health involves taking care of the physical, psychological and spiritual  -self-care enables us to fulfil our aspirations and valuable roles as mothers  -sharing ideas for how to do this with our peers can be helpful |
