# Supplementary material for: Using the COM-B model and Behaviour Change Wheel to develop a theory and evidence-based intervention for women with gestational diabetes (IINDIAGO)
Source: BMC Public Health. 2023 May 15;23:894. doi: 10.1186/s12889-023-15586-y (PMC10186807; doi:10.1186/s12889-023-15586-y)
Supplement: Supplementary file 4 — Additional file 4. Outline of training for lay counsellors. [file 12889_2023_15586_MOESM4_ESM.docx]

**Training lay counsellors in brief behaviour change counselling**

| The training was delivered over 2 days (10 hrs) by three trainers experienced in behaviour change counselling, group work and adult education methods. The approach to the training drew on the philosophy, principles and methods of Motivational Interviewing (Miller and Rollnick, 1991) and Healthy Conversations (Lawrence et al, 2017). Activities were adapted and simplified for the lay counsellors. The initial training was followed by ongoing individual and group coaching and regular group meetings to reflect on practice and to discuss challenges in implementation.  **OUTLINE FOR TRAINING**  **Day 1:** **Introduction to approach and learning discrete skills**  Welcome and introduction  Icebreaker   1. Reflection on past experiences of behaviour change (interactive group activity) 2. Beliefs about why and how people change (group activity) 3. Qualities of a favourite mentor (individual activity and group feedback)   TEA   1. Introduction to philosophy and principles of patient-centred behaviour change counselling (presentation and discussion) 2. Introduction to Stages of Change theory (presentation and paired sharing) 3. Open Discovery Questions (presentation and discussion   LUNCH   1. Active listening practice (paired activity) 2. Formulating Open Discovery Questions (interactive group activity)   CLOSE  **Total: 4.5 hours**  **Day 2: Using skills in context and reflecting on practice**   1. Feedback and reflection on Day 1 2. Comparison of directive and patient-centred counselling styles (video and discussion) 3. Introduction to *Change Ruler* tool   TEA   1. Practice using ODQs and *Change* ruler (paired role play and group feedback) 2. Goal setting exercise using the 4Ws (who, what, where, when)   LUNCH   1. Trainer demonstration to model practice (fishbowl role play and group discussion) 2. Practice with observation, feedback and discussion (role plays with client; counsellor and observer) 3. Resources for education and counselling 4. Summing up, feedback on training, evaluation and next steps   CLOSE  **Total 5.5 hours** |
| --- |
